# Oxidising agents in sub-arc mantle melts link slab devolatilisation and arc magmas

**DOI:** 10.1038/s41467-018-05804-2

**Published:** 2018-08-29

**Authors:** Antoine Bénard, Kevin Klimm, Alan B. Woodland, Richard J. Arculus, Max Wilke, Roman E. Botcharnikov, Nobumichi Shimizu, Oliver Nebel, Camille Rivard, Dmitri A. Ionov

**Affiliations:** 1 Research School of Earth Sciences, The Australian National University, Acton, ACT 2601 Australia; 2 School of Earth, Atmosphere and Environment, Monash University, Clayton, VIC 3800 Australia; 3 Institut für Geowissenschaften, Goethe Universität Frankfurt, 60438 Frankfurt am Main, Germany; 4 Institut für Erd- und Umweltwissenschaften, Universität Potsdam, Karl-Liebknecht-Strasse 24-25, 14476 Potsdam, Germany; 5 Institut für Mineralogie, Leibniz Universität Hannover, Callinstrasse 3, 30167 Hannover, Germany; 6 Institut für Geowissenschaften, Gutenberg Universität Mainz, J.-J.-Becher Weg 21, 55128 Mainz, Germany; 7 Geology and Geophysics Department, Woods Hole Oceanographic Institution, Woods Hole, MA 02543-1052 USA; 8 European Synchrotron Radiation Facility, Grenoble, 38043 France; 9 Géosciences Montpellier, Université de Montpellier and UMR-CNRS 5243, Montpellier, 34095 France; 10 Present Address: Institute of Earth Sciences, Géopolis, CH-1015 Lausanne, Switzerland

## Abstract

Subduction zone magmas are more oxidised on eruption than those at mid-ocean ridges. This is attributed either to oxidising components, derived from subducted lithosphere (slab) and added to the mantle wedge, or to oxidation processes occurring during magma ascent via differentiation. Here we provide direct evidence for contributions of oxidising slab agents to melts trapped in the sub-arc mantle. Measurements of sulfur (S) valence state in sub-arc mantle peridotites identify sulfate, both as crystalline anhydrite (CaSO_4_) and dissolved SO_4_^2−^ in spinel-hosted glass (formerly melt) inclusions. Copper-rich sulfide precipitates in the inclusions and increased Fe^3+^/∑Fe in spinel record a S^6+^–Fe^2+^ redox coupling during melt percolation through the sub-arc mantle. Sulfate-rich glass inclusions exhibit high U/Th, Pb/Ce, Sr/Nd and δ^34^S (+ 7 to + 11‰), indicating the involvement of dehydration products of serpentinised slab rocks in their parental melt sources. These observations provide a link between liberated slab components and oxidised arc magmas.

## Abstract (web summary)

The oxidised nature of arc magmas is either attributed to recycling from the slab or magma differentiation. Here, the authors show that oxidised iron and sulfur, respectively in sub-arc mantle spinel and glass inclusions with elevated U/Th, Pb/Ce, Sr/Nd and δ^34^S, trace dehydration products of slab serpentinites.

## Introduction

The oxidation state of magmas represents the sum of the electron exchange balance between multivalent atoms including major (Fe), minor (Mn), trace (Cr and V) and volatile (S, C and H) elements^1^. The redox conditions, expressed in terms of oxygen fugacity (*f*O_2_), control the evolution and degassing of magmas in volcanic arcs above subduction zones and the formation of ore deposits^2^. In practice, the *f*O_2_ during the generation and evolution of magmas can be estimated on the basis of Fe^3+^/∑Fe (Fe^3+^/(Fe^3+^ + Fe^2+^)) in quenched melts^3^ and olivine-pyroxene-spinel assemblages in crystal-rich rocks^4,5^, or alternatively from S^6+^/∑S (S^6+^/(S^6+^ + S^2−^)) in quenched melts^6–8^.

**Fig. 1:**
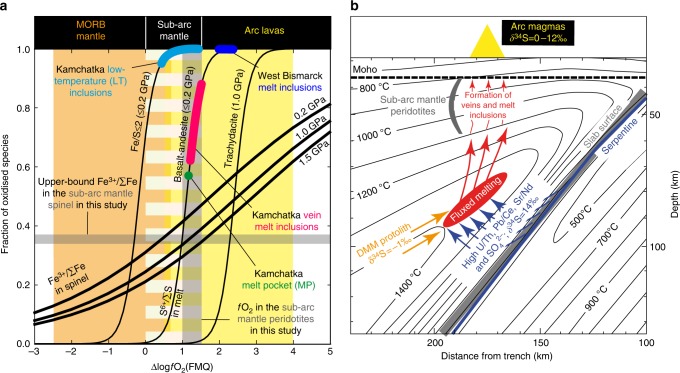
Sulfur valence state at oxygen fugacities prevailing at mid-ocean ridges and subduction zones and the origins of the glass (formerly melt) inclusions (MI) in Kamchatka and West Bismarck sub-arc mantle peridotites. **a** S^6+^/∑S equilibrium (thin black curves) in silicate melts and the range of Δlog*f*O_2_ measured for subduction zone rocks and melts expressed relative to FMQ: orange, abyssal peridotites (MORB mantle)^43,44^; white bars, sub-arc mantle rocks^12,13,34^; yellow, arc lavas^7,8,11^. The S^6+^/∑S equilibrium curves are shown for Fe-poor (Fe/S ≤ 2) and Fe-rich (Fe/S»2) silicate melts at low-pressure ( ≤ 0.2 GPa) conditions^8,16^ and for 1 GPa^40^. The S^6+^/∑S determined by XANES and inferred from Raman spectrometry for Kamchatka LT inclusions, MP, and vein MI and West Bismarck MI are respectively shown in cyan, green, pink and blue. The upper-bound Fe^3+^/∑Fe in spinel measured by EPMA (i.e., in halos next to the inclusions) for the percolated harzburgite samples Av33 (Kamchatka) and 67-02D(7) (West Bismarck) is indicated with a grey horizontal bar (Supplementary Tables 5 and 6). The range of Δlog*f*O_2_ calculated for the percolated harzburgite samples Av33 and 67-02D(7), using the EPMA data on spinel (away from the inclusions and their halos) reported in this study and earlier^26,34^, is indicated with a grey vertical bar (Supplementary Tables 9 and 10). Also shown are the calculated Fe^3+^/∑Fe in spinel in equilibrium with the parental melts of LT inclusions and MP at 0.2, 1 and 1.5 GPa (bold black curves), using the Fe^3+^/∑Fe equilibrium in silicate melts^3^ and partitioning coefficients for Fe^3+^ and Fe^2+^ between Cr-bearing spinel and melt^32^ (Supplementary Table 12). **b** A subduction zone cross-section showing the original position of the studied peridotite xenoliths (‘sub-arc mantle peridotites’) from Kamchatka and West Bismarck in the shallow mantle lithosphere before their ascent in arc magmas. Also indicated are the stability fields for subducted serpentine (antigorite, dark blue line)^50^ and the geochemical features of the inferred fluids derived therefrom (dark blue arrows)^15,50^, DMM (orange)^48,49^ and erupted arc magmas (yellow triangle)^19–21,51^. The inclusions in this study allow probing the redox conditions in and sulfur isotope compositions of the deep mantle wedge (‘fluxed melting’, red field)

In subduction zones, the *f*O_2_ of the different rocks varies over several log units above the fayalite-magnetite-quartz (FMQ) reference buffer assemblage (Fig. 1a). Mid-ocean ridge basalts (MORBs) and abyssal peridotites are representative of the oceanic lithosphere that ultimately undergoes subduction (slab) and are relatively reduced, with *f*O_2_ close to and below FMQ^9^. However, before being subducted, the oceanic lithosphere can be altered and oxidised on or near the seafloor by hydrothermal and metamorphic processes. Arc lavas, generated by interactions between agents derived from the oxidised slab and the relatively reduced mantle wedge, generally display higher *f*O_2_ than MORB, with values above FMQ^7,10,11^. Xenoliths from the sub-arc mantle lithosphere (Fig. 1b), sampled as rock fragments in volcanic deposits, record *f*O_2_ ranging from FMQ to two log units above^12,13^ (Fig. 1a).

The oxidised nature of arcs compared with mid-ocean ridges has been largely attributed to the addition of volatile-rich slab agents to the asthenospheric mantle source in arcs before or during melting^12–16^. Slab-derived S^6+^, generally linked with oxygen to form reactive sulfate (SO_4_^2−^) ions, has been proposed as a key player in mantle wedge oxidation^2,16–18^, as it is a powerful oxidising agent able to convert 8 mol of Fe^2+^ into Fe^3+^ per mole of S^6+^ according to the reaction:

1 $${\mathrm{SO}}_4^{2 - } + {\mathrm{8FeO < = > S}}^{{\mathrm{2 - }}} + {\mathrm{4Fe}}_{\mathrm{2}}{\mathrm{O}}_{\mathrm{3}}$$

Mounting evidence from stable isotope measurements on deeply subducted rocks^15,17,18^ supports the idea that fluids released from these rocks could be enriched in SO_4_^2−^ (Fig. 1b). In parallel, the involvement of slab-derived S in the generation of arc magmas is supported by S isotope systematics, with elevated δ^34^S in arc melts and volcanic gases interpreted as the result of S recycling from the slab^19–21^ (Fig. 1b). However, it remains unclear if the released slab fluids are sufficiently abundant and oxidised to travel tens of kilometres to the zones of arc melt generation without being reduced by the ambient mantle (Fig. 1b). Indeed, direct evidence for the presence of recycled, slab-derived sulfate in sub-arc mantle melts sampled at their earliest stages of evolution remains to be found.

The need for identifying such a missing link also stands out, because the oxidised nature of the sources of arc magmas has been disputed on the grounds that the similar V/Sc and Fe/Zn measured in ridge- and arc-associated basalts may imply comparable *f*O_2_ in the magmatic source regions of both tectonic settings^22–24^. A corollary to this interpretation is that the more oxidised nature observed for arc magmas compared to MORB must result from secondary processes occurring during magma ascent^22–24^. Resolution of this problem has global geochemical significance for quantifying mass fluxes of redox-sensitive elements (e.g., Fe and S) through subduction zones and for the origin of related, S-bearing ore deposits^2^.

Here we report Fe and S valence state and S isotope measurements on sub-arc mantle peridotite xenoliths and their spinel-hosted glass (formerly melt) inclusions (MIs) and pockets, which record the in situ oxidation state of primitive arc melts as they ascend to the overlying crust. The results show that large quantities of S^6+^ are transferred from the slab to magma generation zones in the mantle wedge, and that sulfate contributes to the oxidation of Fe in sub-arc mantle minerals and the formation of magmatic Cu-rich sulfide.

## Results

### Samples

The investigated samples are all spinel harzburgite xenoliths from the sub-arc mantle lithosphere; these peridotites record relatively low temperatures ranging from 650 to 1000 °C, as calculated using olivine-spinel thermometry^25–27^ (Fig. 1b). The mantle xenoliths were brought to the surface by recent volcanic activity at the Avacha (samples Av24, Av25 and Av33) and Ritter (sample 67-02D(7)) volcanoes, respectively, located in the Kamchatka (Russia) and West Bismarck arcs (Papua New Guinea)^26–29^.

**Fig. 2:**
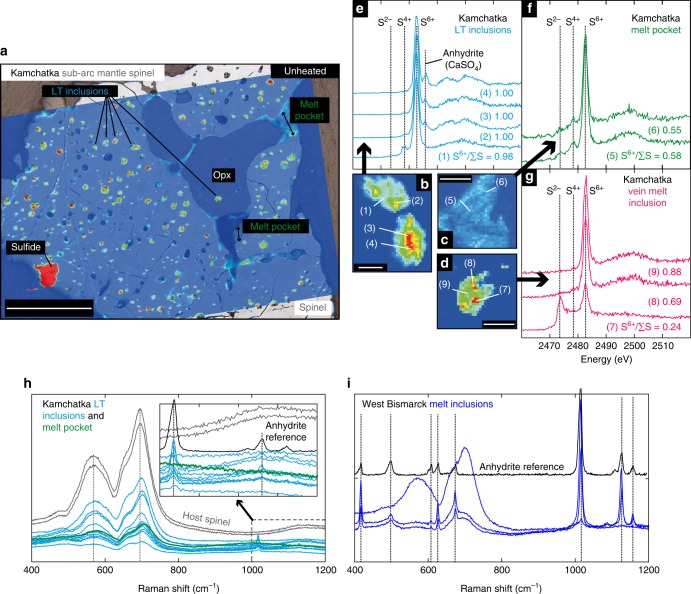
Spinel-hosted glass (formerly melt) inclusions (MI) from the mantle lithosphere beneath the Kamchatka and West Bismarck arcs and their sulfur distribution and valence state. **a** A reflected-light image of a spinel grain containing many LT inclusions and MP. Coloured inlay in **a** shows the elemental distribution of S in the spinel, LT inclusions and MP determined by S Kα X-ray fluorescence imaging (red, high S concentration; blue, low S concentration). Note the presence of a LT inclusion in an orthopyroxene (Opx) from the host harzburgite, which supports the formation of these inclusions by pervasive percolation of their parental melts in the sub-arc mantle lithosphere. Scale bar length is ~200 µm. **b**–**d** Uncalibrated S Kα X-ray fluorescence maps of **b** LT inclusions, **c** MP and **d** vein MI. The LT inclusions and vein MI are characterised by a heterogeneous distribution of sulfur. The S-free spinel appears in dark blue. Scale bar lengths are respectively ~10 µm in **b** and ~20 µm in **c**, **d**. **e**–**g** S K-edge XANES spectra of **e** LT inclusions, **f** MP and **g** vein MI. Spectra numbers refer to the spot positions shown in **b**–**d**. Vertical black lines indicate energies for specific S valence states: 2482.8 eV for S^6+^, 2478 eV for S^4+^ and 2472.5 eV for S^2−^ in crystalline Fe-S compounds. The additional feature at ~2486 eV is typical for crystalline anhydrite (CaSO_4_). Some S^4+^ is generated during XANES measurements of glasses by photo-reduction of S^6+^. The LT inclusions contain solely S^6+^ in glass and in anhydrite. The MP contain S^2−^ and S^6+^ in glass with 0.55 ≤ S^6+^/∑S ≤ 0.58. The vein MI dominantly contain S^6+^ in the glass with S^6+^/∑S up to 0.88 but also show varying contributions of S^2−^ from immiscible sulfides (Supplementary Table 1). **h**, **i** Raman spectra for **h** multiple LT inclusions and an MP, and **i** West Bismarck MI, compared with their host spinel (grey spectrum) and an anhydrite (CaSO_4_) reference (black spectrum). All these inclusions, with the exception of the MP, contain anhydrite. Note that all the inclusions for which data are presented here are unheated

As originally described in detail in Ionov et al.^28^ and Bénard et al.^29^, Cr-bearing spinel in Kamchatka xenoliths contains glass MIs with compositions spanning andesite-dacite (‘LT inclusions’, where LT stands for low temperature), magnesian andesite (melt pockets, ‘MP’) and dacite-rhyolite of boninitic affinity (‘vein MIs’). Spinel in West Bismarck xenoliths contains magnesian andesite glass (‘West Bismarck MIs’, Supplementary Fig. 1). The Mg# (Mg/(Mg + Fe_t_), where Fe_t_ means all Fe is treated as Fe^2+^) of the inclusion glasses is generally ≥ 0.6 in MP, vein MI and West Bismarck MI but is < 0.4 in LT inclusions (Supplementary Table 1). High concentrations of slab agents in the inclusion parental melts were inferred from lithophile trace element signatures and very high volatile abundances in the glasses^28–31^; the second compositional feature (e.g., up to ~10 wt% H_2_O and ~1 wt% CO_2_, ref. 29) also indicates that melt entrapment occurred at high pressure in the sub-arc mantle lithosphere (Fig. 1b). The inclusion parental melts are ‘exotic’ liquids (i.e., they were trapped on their way to the surface and not formed in situ), which were confined in spinel in the sub-arc mantle lithosphere during a percolation process, either pervasive (LT inclusions, MP and West Bismarck MI) or along channels (vein MI)^28,29^ (Figs. 1b and 2a). More detail on the spinel-hosted MI investigated in this study is provided in the Supplementary Discussion.

In contrast with earlier works on Kamchatka inclusions^28–31^, we have not used experimentally treated (i.e., heated) MI and their host spinel grains to investigate S and Fe valence states in this study. This choice was made considering the possibility of an Fe^3+^–Fe^2+^ exchange reaction during experiment between melt and spinel, in which Fe^3+^ is compatible^32^, or Fe^3+^ oxidation in melt triggered by H^+^ loss from the inclusions through either diffusion^33^ or leakage. Instead of using treated MI, we have investigated unheated inclusions that are found naturally free of daughter silicate phases^28,29^ (Supplementary Figs 2–4 and Discussion).

### Sulfur abundances and valence state

The full dataset supporting this study is given in Supplementary Tables 1–13. Micro X-ray fluorescence (XRF) mapping combined with micro X-ray absorption near-edge structure (XANES) spectroscopy show that sulfur is heterogeneously distributed in LT inclusions and vein MI, while MP have more homogeneous S concentrations (Fig. 2a–d). The ‘bulk’ glasses in Kamchatka and West Bismarck MI contain 150–6000 p.p.m. S (Supplementary Tables 1–3). The molar Fe/S of the inclusion glasses, an important feature regarding S dissolution mechanism in hydrous silicate melts^16^, mostly ranges from 8 to 20 in MP, vein MI and West Bismarck MI, but only from 1 to 2 in LT inclusions (Supplementary Tables 1–3).

All spinel-hosted inclusions contain S^6+^ present as SO_4_^2−^ within the glass structure (Fig. 2e–g). The LT inclusions contain almost solely S^6+^ in the glass (S^6+^/∑S always close or equal to 1) and often exhibit the contribution of S^6+^ from crystalline anhydrite (CaSO_4_, Fig. 2d and Supplementary Fig. 5). The MP glass contains a homogenous mixture of dissolved S^6+^ and S^2−^ with S^6+^/∑S~0.57 (Fig. 2e). In addition to S^6+^, some XANES spectra of vein MI indicate the presence of S^2−^ that is heterogeneously distributed as a S-bearing solid phase (i.e., an immiscible sulfide, Fig. 2d, g and Supplementary Fig. 5). Depending on the contribution of the immiscible sulfide to the overall XANES spectral measurement, the calculated S^6+^/∑S for ‘bulk’ vein MI are variable and can be as low as 0.24–0.44 (Fig. 2d, g and Supplementary Fig. 5). The highest S^6+^/∑S for vein MI range from 0.62 to 0.88 in glassy areas displaying a more homogeneous S distribution (Fig. 2d, g and Supplementary Fig. 5), which indicate the presence of dissolved S^2−^ in the glass of these inclusions as well. Further characterisation of multiple inclusions using Raman spectrometry reveals that anhydrite is systematically present in LT inclusions (Fig. 2h) and West Bismarck MI (Fig. 2i).

### Imaging and mapping

The presence of sub-micrometre anhydrite crystals in LT inclusions and West Bismarck MI is one of the most important results of this study and is further documented by imaging and mapping using electron probe micro-analysis (EPMA) and scanning electron microscope (SEM) techniques (Supplementary Figs 2–4). Although not evidenced by any XANES measurement, rare sulfide blebs sometimes occur in LT inclusions (Supplementary Figs 2–4, 6 and 7). An important observation is that these sulfides may contain as much as 20 wt% Cu (Supplementary Table 4).

### Iron valence state

**Fig. 3:**
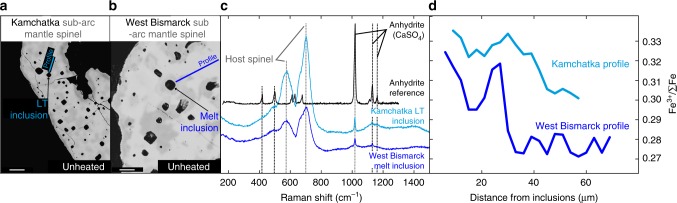
Coexisting anhydrite in glass (formerly melt) inclusions (MI) and increased ferric iron abundances in their host spinel from the mantle lithosphere beneath Kamchatka and West Bismarck arcs. Back-scattered electron (BSE) images of chemical zoning (halos) of **a** Kamchatka and **b** West Bismarck inclusion-bearing mantle spinel. Scale bar lengths are respectively ~50 µm in **a** and ~20 µm in **b**. **c** Raman spectra of the inclusions (LT inclusions and West Bismarck MI) in **a**, **b** plus an anhydrite (CaSO_4_) reference (black spectrum). **d** Measured Fe^3+^/∑Fe profiles in spinel adjacent to the inclusions indicated with lines in **a**, **b**. The variable contrast of spinel visible in the BSE images in **a**, **b** is related to changes in the concentrations of Al, Cr, Fe and Mg in irregular halos surrounding anhydrite-bearing MI (Supplementary Tables 5 and 6). Note that all inclusions for which data are presented here are unheated

Measurements of Fe valence state in the MI-hosting spinel using EPMA calibrated with secondary (Fe^3+^, Fe^2+^)-bearing spinel standards^4^ reveal Fe^3+^ zoning in irregular halos^28^ next to the inclusions (Fig. 3a–d and Supplementary Tables 5–7). The highest Fe^3+^/∑Fe in spinel is found adjacent to anhydrite-bearing inclusions and decreases away from the MI, typically from 0.34 to 0.30 and 0.33 to 0.27 for LT inclusions and West Bismarck MI^34^, respectively (Fig. 3d and Supplementary Tables 5 and 6). The halos are also characterised by significantly lower Cr and higher Al concentrations (by up to 0.10–0.25 At%), but comparatively little variations in Fe^2+^, Mg and Mg# (by only 0.01–0.04) than in spinel away from the inclusions (Supplementary Tables 5 and 6); these halos are very similar to those around MP reported by Ionov et al.^28^ (Supplementary Fig. 8 and Supplementary Table 8). Further EPMA measurements reveal that the Fe^3+^/∑Fe increase can be also restricted to the LT inclusion-bearing rims of some spinel grains that are only partially impregnated with melt (Supplementary Fig. 7 and Supplementary Table 5).

### Oxygen fugacity estimates

The *f*O_2_ values (expressed as the deviation from FMQ in log units, Δlog*f*O_2_) inferred from the S^6+^/∑S equilibrium in ‘Fe-poor’ silicate melts (Fe/S ≤ 2) at ≤ 0.2 GPa^16^ range from FMQ + 0.5 to FMQ + 1.5 for LT inclusions. The Δlog*f*O_2_ values inferred from the S^6+^/∑S equilibrium in ‘Fe-rich’ silicate melts (Fe/S»2) at ≤ 0.2 GPa^8,16^ range from FMQ + 1 to FMQ + 1.5 for MP and vein MI, and correspond to ca. FMQ + 2 for West Bismarck MI (Fig. 1a). It is noteworthy that as anhydrite saturation is evidenced in all LT inclusions and West Bismarck MI investigated in this study (Fig. 2e, h, i and Supplementary Fig. 5), the Δlog*f*O_2_ inferred from the S^6+^/∑S equilibrium for these inclusions may constitute only lower-bound estimates. Calculations at 1.5 GPa with a thermometer^5^ and an oxybarometer^9^ for the MI-bearing harzburgite samples, using the stable compositions of spinel measured away from the inclusions and their halos^34^ (Supplementary Tables 5 and 6) and those of coexisting silicate minerals previously reported by Bénard et al.^26^, yield Δlog*f*O_2_ ranging from FMQ + 1.0 to FMQ + 1.4 ( ± 0.2–0.3, Fig. 1a and Supplementary Tables 9 and 10).

### Sulfur isotope compositions

From the absence of an immiscible S-bearing phase, and the homogeneous elemental distribution and valence state of S dissolved in their glasses (Fig. 2c, f), it appears that MP are the best suited for the determination of pristine δ^34^S using secondary ion mass spectrometry (SIMS). Furthermore, the other types of inclusions are much smaller than MP in size^28^ (Fig. 2a), which render SIMS analysis very difficult. We have further selected bubble-free MP found in the harzburgite spinel for these measurements. The δ^34^S (see Methods for the usage of delta notation) measured by SIMS ranges from + 7 to + 11‰ ( ± 1–1.6, 2*σ*) in the glass of MP (Supplementary Table 11).

## Discussion

Before interpreting further the origins of elevated *f*O_2_ in sub-arc mantle melts and harzburgites, it should be considered if post-entrapment processes affected this feature in the studied inclusions or not. One of these processes is H_2_ loss from the inclusions, which can displace the equilibrium:

2 $${\mathrm{2FeO}} + {\mathrm{H}}_{\mathrm{2}} {\mathrm{O}} \, < {=} > \, {\mathrm{Fe}}_{\mathrm{2}} {\mathrm{O}}_{\mathrm{3}} + {\mathrm{H}}_{\mathrm{2}}$$

to the right side as H_2_O dissociates according to:

3 $${\mathrm{H}}_{\mathrm{2}}{\mathrm{O}}\, < {=} > \, {\mathrm{H}}_{\mathrm{2}} + {\mathrm{1/2O}}_{\mathrm{2}}$$

and H_2_ escapes from the system. The very low totals of EPMA analyses in Kamchatka MI (down to 88 wt% for LT inclusions, 98 wt% for MP and 92 wt% for the vein MI, Supplementary Table 1) indicate bulk volatile contents close to the maximum H_2_O abundances previously determined by SIMS analysis, with up to ~12 wt% and ~3 wt%, respectively, in LT inclusions and MP^30,31^, and ~10 wt% H_2_O in the vein MI^29^. The EPMA setup used in this study has been previously demonstrated to provide deficiency of totals of analyses in good agreement with SIMS volatile data^29^. Therefore, the inclusions in this study are among the most volatile-rich identified in these sub-arc mantle xenoliths so far and, as such, unlikely suffered appreciable H_2_O loss.

Insignificant H_2_O loss from the inclusions is further suggested by the fact that solid-state diffusion of H^+^ within spinel is also two to three orders of magnitude slower than in olivine^35,36^. With the relatively low temperatures recorded in the percolated harzburgites in this study (950–1020 °C, Supplementary Table 10) and prevailing in the shallow mantle below the Kamchatka and West Bismarck arcs^25–27^, H^+^ diffusivities of ≤ 10^−14^ m^2^ s^−1^ are expected in spinel^36^. Only 30 min were inferred for the ascent from the sub-arc mantle of West Bismarck peridotite xenoliths from the same sampling sites as in this study^37^. Several hours have been calculated for the ascent of mantle xenolith-bearing magmas at convergent margins with thicker continental crust^38^, such as is the case for the Kamchatka arc. We conclude that volatile loss has not significantly affected the H_2_O-rich inclusions during the fast ascent of the xenoliths to the surface.

The calculated *f*O_2_ for the inclusions and their host harzburgites in this study fall within the range typically observed for subduction zone lavas and mantle rocks, respectively (Fig. 1a). All Δlog*f*O_2_ estimated above for the inclusions may be shifted even further up if one considers that increasing pressure has a significant effect on the S^6+^/∑S equilibrium in silicate melts^39,40^. However, quantifying this pressure effect remains difficult in the present state of knowledge, as it likely interplays in a complex way with those imposed by the variations in melt composition (e.g., Fe/S, ref. ^16^). For instance, Moretti and Baker^39^ have modelled a shift of the S^6+^/∑S equilibrium of only ca. + 0.5 log units in *f*O_2_ for hydrous tholeiite melts, whereas Matjuschkin et al.^40^ have experimentally inferred a shift of ca. + 1.5 log units for hydrous trachydacite melts (Fig. 1a), both with a pressure increase of ~1 GPa.

The magnitude of the pressure effect on the S^6+^/∑S equilibrium for the inclusions in this study can be tested independently using the Fe^3+^/∑Fe equilibrium in silicate melts^3^ and the experimentally determined partitioning coefficients for Fe^3+^ and Fe^2+^ between Cr-bearing spinel and melt^32^. The upper-bound Fe^3+^/∑Fe measured in sub-arc mantle spinel adjacent to the MI (0.34–0.37, Supplementary Tables 5 and 6), correspond to those calculated for Δlog*f*O_2_ ranging from FMQ + 1 to FMQ + 1.5 at 1–1.5 GPa in spinel in equilibrium with the parental melts of LT inclusions and MP (Fig. 1a and Supplementary Table 12). As this Δlog*f*O_2_ range corresponds well to the results of oxybarometric calculations for the host sub-arc mantle harzburgites in this study (Fig. 1a and Supplementary Table 10), we conclude that it constitutes the best estimate for the formation of their inclusions. Furthermore, the calculated Fe^3+^/∑Fe in spinel provides additional evidence that the formation of the inclusions occurred at ≥ 1 GPa, i.e., in the sub-arc mantle (Fig. 1a, b). This test based on our EPMA data does not preclude an effect of pressure on the S^6+^/∑S equilibrium in silicate melts, but rather suggests that it is within the error of the Δlog*f*O_2_ estimates (typically ± 0.5, Supplementary Table 10) for the samples in this study.

**Fig. 4:**
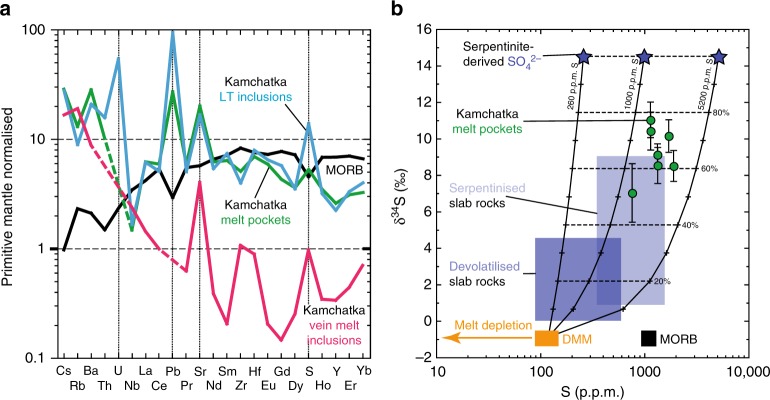
Lithophile trace element and sulfur isotopic compositions of the studied glass (formerly melt) inclusions. **a** Primitive mantle-normalised^66^ lithophile trace element abundances (averages, Supplementary Table 13) in the glasses^28,29^ of Kamchatka LT inclusions (cyan pattern), MP (green pattern) and vein MI (pink pattern) compared to MORB^46,48,49^ (black pattern). Sulfur is always enriched in comparison with lithophile trace elements as incompatible as S^2−^ (e.g. Dy, ref. ^67^) in the inclusion glasses. This further suggests that S was enriched and/or oxidised in the mantle sources of the inclusion parental melts, since S^6+^ is much more soluble than S^2−^ in silicate liquids^8,58^. **b** A δ^34^S (‰) vs. S content (p.p.m.) plot for MP in this study (green dots, Supplementary Table 11), compared with DMM estimates^46,48,49^ (orange field) and MORB data^48,49^ (black field). The error bars represent the 2*σ* analytical uncertainty on the δ^34^S values reported in this study (Supplementary Table 11). Also shown are high-pressure slab rocks^15^ (light and dark blue fields) and black mixing lines between a DMM source and agents derived from subducted serpentinite (dark blue stars), as inferred from high-pressure slab rocks^15^, with variable bulk S contents (260–5200 p.p.m., exclusively present as sulfate). These variable bulk S contents are calculated to account for different dilution factors of S in agents derived from subducted serpentinite, depending on whether these agents contain only H_2_O and S (5200 p.p.m. S) or more components ( < 5200 p.p.m. S). For this purpose, H_2_O (5 wt%) and S (260 p.p.m.) abundances inferred to be lost by subducted serpentinite^15^ are used. The composition of the volatile fraction in the parental melts of MP (S abundances and δ^34^S) is consistent with mixing between DMM and subducted serpentinite-derived agents containing ≥ 1000 p.p.m. sulfate (SO_4_^2−^). Given that the mantle source protoliths of the MP parental melts are more depleted than DMM (**a**), 1000 p.p.m. S in agents derived from subducted serpentinite likely represents a lower bound and the mixing lines could be shifted to the left in **b** (‘melt depletion’, orange arrow). Note that all MP for which S data are presented here are unheated

In situ observations demonstrate that sulfate is present in melts from the mantle below the Kamchatka and West Bismarck arcs, recording Δlog*f*O_2_ of at least ca. FMQ + 1 (Fig. 1a). The mantle source protoliths (i.e., considered before fluxing by slab agents occurs in the mantle wedge, Fig. 1b) of the inclusion parental melts must be more depleted than the MORB source mantle (DMM), as inferred from their relatively low heavy and middle rare-earth element and Y abundances^28,29^ (Fig. 4a). For instance, ~15% melt extraction from DMM was calculated for the mantle source protoliths of the boninitic parental melts of the vein MI^29^. Studies of primary MORB show that these magmas are equilibrated in their sources at FMQ^41,42^, whereas the *f*O_2_ of abyssal peridotites dominantly ranges below FMQ^43,44^ (Fig. 1a). Therefore, it is unlikely that the original *f*O_2_ is higher than FMQ in the more depleted mantle source protoliths of the inclusion parental melts in this study, owing to the preferential decrease in Fe^3+^ concentrations and Fe^3+^/∑Fe in the mantle with increasing melt depletion^32,34,45^.

Furthermore, 15% melting of a DMM source containing 90–150 p.p.m. S (ref. ^46^) and the formation of a basalt dissolving ≥ 1000 p.p.m. S at ≤ 1.5 GPa and ≥ 1200 °C (ref. ^47^) is also unlikely to leave significant amounts of residual sulfides. However, all inclusion types in this study are particularly enriched in S and other volatiles^29–31^ (Fig. 4a, Supplementary Tables 1–3 and Discussion). The high δ^34^S measured in the MP are not intrinsic to the upper Earth’s mantle such as DMM^48,49^, but are inevitably related to recycled components (Fig. 4b). It has been recently shown that subducted serpentinite is a possible carrier of large amounts of sulfate with elevated δ^34^S to sub-arc mantle depths^15,17,18^. A contribution from subducted serpentinite is further consistent with the very high U/Th, Pb/Ce and Sr/Nd of the inclusion parental melts in this study^50^ (Fig. 4a and Supplementary Table 13). Mixing models show that the high S contents and δ^34^S in MP (i.e., representative of the composition of the volatile fraction dissolved in their parental melts, Fig. 4b) require the involvement of subducted serpentinite-derived agents carrying ≥ 1000 p.p.m. S as sulfate (with an assumed δ^34^S of 14.5‰ in the original agents^15^). Therefore, high S, S^6+^/∑S and δ^34^S in melts percolating through the mantle below the Kamchatka and West Bismarck arcs must result predominantly from the addition of S-rich, oxidised slab agents to their depleted source protoliths in the mantle wedge (Fig. 1b). As the results in this study link the presence of sulfate recycled from the slab and elevated δ^34^S in sub-arc mantle melts, by extension, they also support the idea that high δ^34^S in primitive, un-degassed lavas from global arcs^19–21,51^ (Fig. 1b) can serve as a reliable proxy for the presence of oxidising slab agents in their mantle wedge sources.

The lower-bound Δlog*f*O_2_ inferred for the MP in this study (ca., FMQ + 1, Fig. 1a) precisely corresponds to previous estimates based on the Fe^3+^/∑Fe in spinel from primitive arc lavas^11^. In this context, it is interesting to note that the major element compositions of the original parental melts of MP and vein MI (respectively, magnesian andesite^28^ and high-Ca boninite^29^) are close to those of mantle-derived liquids typically found at subduction zones, whereas LT inclusions rather resemble low-temperature hydrous melts produced in partial melting experiments of slab rock analogues^52,53^ (Supplementary Table 1). This dichotomy in the apparent origins of the inclusion parental melts is directly illustrated by the variable Mg# of their glasses, which are close to those for melts in equilibrium with the mantle in MP and vein MI but much lower in LT inclusions (Supplementary Table 1). Note that, as variations in the Mg# of spinel next to these inclusions are very limited (Supplementary Tables 5 and 8), it is improbable that this parameter was significantly modified in the included melts by post-entrapment processes (e.g., re-equilibration through solid-state diffusion in spinel). Therefore, the results in this study suggest that slab agents transporting recycled sulfate ions can not only maintain their oxidising capacity during kilometre-scale percolation in the mantle wedge, but this oxidised sulfur can be effectively transferred to primitive arc magmas during mantle melting (Fig. 1b).

Considering the percolated harzburgite samples in this study, their calculated equilibrium *f*O_2_ fits within the upper range previously reported for sub-arc mantle peridotites^13,34^ (Fig. 1a). Recently, elevated ∆log*f*O_2_ and orthopyroxene contents in SiO_2_-rich, sub-arc mantle harzburgite xenoliths have been both related to fluxed melting processes involving oxidised slab agents during their formation^26,34^. In this study, it is shown that ≥ 1000 p.p.m. of sulfate from subducted serpentinite can contribute to the volatile fraction in the mantle-derived parental melts of MP (Fig. 4b), while SiO_2_-rich liquids resembling hydrous slab melts in LT inclusions contain ~3400 p.p.m. sulfate on average (Supplementary Table 1). On the one hand, a SiO_2_-rich melt is required to explain the generation of an orthopyroxene-rich sub-arc mantle through the reaction:

4 $${\mathrm{Olivine}} + {\mathrm{SiO}}_{2}\, < {=} > \,{\mathrm{orthopyroxene}}$$

during fluxed melting^26^. On the other hand, ~3000 p.p.m. sulfate are required to elevate ∆log*f*O_2_ from FMQ − 0.5 to FMQ + 1.5 through reaction (1) in a primary melt containing ~10 wt% total FeO (ref. ^15^). An elevation of ∆log*f*O_2_ from FMQ-0.5 to FMQ + 1.5 overlaps with the shift between the average upper-bound oxidation states recorded in abyssal and sub-arc mantle peridotites, respectively^13,34,43,44^ (Fig. 1a).

Detailed characterisation of the percolated harzburgite samples in this study allows further insights into melt-rock interaction processes occurring in the sub-arc mantle. Ionov et al.^28^ originally attributed the formation of LT inclusions and MP to the pervasive percolation of ‘exotic’ melts in the sub-arc mantle lithosphere. This is in line with the generally (Cr, Fe^3+^, Fe^2+^)-rich nature of spinel in the percolated harzburgite samples, in comparison with those recognised as ‘pristine’ residues and found at the same sampling sites^25–29,34^. The observation that the Cr, Fe_t_ and Fe^3+^/∑Fe increase is sometimes restricted to the LT inclusion-bearing rims of some spinel grains further shows that this mineral experienced oxidation because of reactions with sulfate-bearing melts originally percolating at grain boundaries (Supplementary Fig. 7 and Supplementary Table 5). Therefore, melt-peridotite interactions in the sub-arc mantle lithosphere have partially or entirely modified the chemistry of spinel grains, likely through dissolution and re-precipitation of this mineral (Supplementary Discussion). This process is consistent with the inferred redox equilibrium between the percolated harzburgite samples and the inclusion parental melts at ∆log*f*O_2_ ranging from FMQ + 1 to FMQ + 1.5 (Fig. 1a and Supplementary Discussion).

Melt-peridotite interactions in the sub-arc mantle lithosphere were constrained by diffusion to occur only several months before eruption in the case of some West Bismarck peridotite xenoliths from the same sampling sites as in this study^37^. However, it is unlikely that these timescales are short enough to fully prevent post-entrapment Fe^2+^–Mg re-equilibration between the included melts and spinel, for instance during cooling^54,55^. The small extent of this process, however, is traced by the presence of irregular halos with very limited variations in Fe^2+^, Mg and Mg# around the inclusions in this study (Fig. 3d, Supplementary Tables 5, 6 and Discussion). The LT inclusions and MP analysed in this study do not contain daughter silicate phases; the former only host anhydrite and rare sulfides^28^ (Figs. 2, 3 and Supplementary Fig. 4). Therefore, if chemical variations in spinel halos are related to post-entrapment crystallisation from the inclusion parental melts, they can only involve these S-bearing phases. Simple chemical exchange reactions between a sulfate-bearing melt and spinel can be inferred from the variations in atomic concentrations within the EPMA profiles around the inclusions (Supplementary Tables 5, 6 and Discussion). From these exchange reactions, it appears that higher Fe^3+^/∑Fe in the halos can only originate from the oxidation of small amounts of Fe^2+^ from the host spinel by S^6+^ present in the inclusion parental melts through reaction (1), or a similar one involving H_2_SO_4_ and FeS compounds (Supplementary Discussion). We conclude that the (Cr, Fe^3+^, Fe^2+^)-rich nature of spinel in the percolated harzburgite samples, further substantiated by the presence of Fe^3+^-rich halos, primarily traces S^6+^–Fe^2+^ redox coupling during melt-rock interactions within the sub-arc mantle lithosphere. Therefore, it is directly shown in this study that sub-arc mantle oxidation can proceed during melt-rock interactions involving SiO_2_-rich and S^6+^-bearing melts (Fig. 3d & Supplementary Fig. 7). These observations, made here in xenoliths from the sub-arc mantle lithosphere, provide an analogue for the fluxed melting reactions typically occurring throughout the mantle wedge^26,34^ (Fig. 1b).

Melt-rock interactions involving S^6+^–Fe^2+^ redox coupling can eventually lead to the formation of sulfide species according to reaction (1) (Supplementary Figs 2–4, 6 and 7). These sulfides, when present as an immiscible phase in LT inclusions, are typically enriched in Cu at the contact with the enclosed, sulfate-bearing silicate melts (Supplementary Fig. 4 and Supplementary Table 4), which is consistent with the composition of sulfides formed at high *f*O_2_ in experiments^56^. This compositional feature of sulfides trapped in the inclusions contrasts with those found disseminated in the host spinel harzburgite, which are dominated by (Fe, Ni)-bearing mono-sulfide solid solution, pyrrhotite and pentlandite species with systematically < 1 wt% Cu (ref. ^57^). A similar metal enrichment process in sulfides formed under oxidised conditions has been suggested for some other precious metals such as gold^58^. Therefore, our results provide in situ evidence for the role of oxidised conditions in the concentration of metals of economic interest in immiscible, S^2−^-bearing phases at subduction zones.

Overall, as this study directly documents the oxidised nature of melts from the Kamchatka and West Bismarck arcs at mantle conditions (Fig. 1b), it rules out melt differentiation in the arc crust as a cause for this oxidation. Instead, slab-derived sulfate originating from δ^34^S- and (U, Pb, Sr)-rich subducted serpentinites^15,50^ is demonstrated to be a potentially oxidising agent added to mantle wedge magma sources. Inevitably, this implies that slab agents can maintain their oxidising capacity during migration through the lower mantle wedge (Fig. 1b), and hence they are able to deliver oxidised volatile species to the source regions of arc magmas and affect the ‘redox budget’ in subduction zones.

## Methods

### XRF maps and XANES

XRF maps and XANES spectra at the S K-edge were collected at the European Synchrotron Radiation Facility (ESRF; Grenoble, France) using the scanning X-ray microscope of the ID21 beamline and a micro-focused beam. The beamline uses a Si (111) double-crystal mono-chromator and the energy was calibrated to the position of the white line of gypsum (2482.94 eV). The incident beam intensity was measured using a photodiode. All measurements were performed in fluorescence mode with a focused beam of 0.4 × 0.8 µm because of the small size of most of the melt inclusions ( ≤ 50 µm) investigated in this study. A focussed beam in combination with a high photon flux may cause damage to the sulfur-bearing glass samples, generating S^4+^ by photo-reduction^59–61^. In order to minimise the focussed beam damage, an attenuator of Al foil with 6 µm thickness was applied to reduce the incident flux on the sample. Before each XANES measurement, the melt inclusion was localised within the spinel by simultaneous Si, Al and S Kα XRF imaging with step sizes of 1 or 2 µm and counting times of 1 s per step. Results of the S Kα X-ray maps are shown in Fig. 2 and Supplementary Fig. 5.

The XANES spectra were acquired by continuously scanning the mono-chromator and changing the gap of the undulator with a step size of 0.23 eV. One to 10 quick scans (with the acquisition time fixed at 0.1 s per energy step) were collected and stacked to reduce signal-to-noise ratio. The background of the spectra was subtracted by fitting the energy region below the edge with a quadratic function and subtracting this from the spectra. The edge jump was normalised to unity by fitting an arctangent and a Gaussian function to the spectra. The S^6+^/∑S was calculated using integrated intensities collected on energy windows corresponding to S^2−^ and S^6+^ following the method of Jugo et al.^8^ and Wilke et al.^60^. The XANES spectra and calculated S^6+^/∑S are shown in Fig. 2 and Supplementary Fig. 5, and given in Supplementary Table 1.

### Raman spectrometry

Raman spectra were collected with a Renishaw micro-Raman spectrometer (RM-1000) using the 532 nm line of a Nd:YAG laser at the Goethe University (Frankfurt am Main, Germany)^61^. The spectrometer was equipped with a Leica DMLM optical microscope and a Peltier-cooled, charge-coupled device detector. All spectra were collected from polished (~120 µm thick) sections and separated spinel grains mounted into epoxy in the wavenumber range 100–3000 cm^−1^. The spectral resolution was 2 cm^−1^ and a quasi-backscattering geometry was employed. Exposure times were 60 s with the laser power fixed at 10–20%. No effect of laser beam exposure (i.e., beam damage) on the qualitative results were observed with these analytical conditions; for instance, only variations in signal-to-noise ratio are observed for lower acquisition times. The system was calibrated using the 519 cm^−1^ band of a silicon wafer^62^. The calibration was checked with a Ne lamp^63^. The wavenumber accuracy was around ~1 cm^−1^. The Raman spectra are shown in Figs. 2, 3 and Supplementary Fig. 6 with the compositions of the investigated inclusions given in Supplementary Tables 1–3.

### EPMA analyses of the inclusion glasses

The major element compositions and sulfur contents of the inclusion glasses were determined by EPMA at the Research School of Earth Sciences (RSES) of the Australian National University (ANU; Canberra, Australia). The instrument used was a Cameca SX 100 operating at an accelerating voltage of 15 kV, defocused beams of 5–20 µm in diameter and reduced beam currents of 2–10 nA. Counting times were 5–10 s on background and 20–30 s (Cr and Ni), 15 s (Ca and Ti) and 10 s for all other elements on peaks, with Na and K analysed first. Sulfur was independently analysed in peak integral mode using three spectrometers (respectively with a pentaerythritol and two large pentaerythritol crystals), with increased sample currents and counting times. Matrix effects were corrected using the Phi (r) z modelling available on Peak Sight^©^ software from Cameca^TM^.

These analytical conditions allowed minimising alkali metal losses and/or migration from the analysed area; this setup was previously used to report Na_2_O concentrations of up to 6 wt% in Kamchatka sub-arc mantle MI^28,29^. In addition, this setup has been previously demonstrated to provide deficiency of totals of analyses in good agreement with SIMS volatile data^29^.

### EPMA and SEM imaging and mapping

EPMA and SEM maps and quantitative analyses were respectively acquired at RSES and the Centre for Advance Microscopy at ANU. The instruments used were the Cameca SX 100 (EPMA, operating at an accelerating voltage of 15 kV and a beam current of 40 nA) and a Schottky Field Emission Hitachi 4300 SE/N (SEM, operating at the same conditions as the EPMA).

### EPMA analyses of spinel with Mössbauer standards

The Fe^3+^/∑Fe of spinel was determined by EPMA at the Goethe University using a set of secondary spinel standards following the procedure outlined by Wood and Virgo^4^. The analyses were performed with a JXA-8900 superprobe, using six spinel standards for which Fe^3+^/∑Fe had been previously determined by Mössbauer spectroscopy by Wood & Virgo^4^. Measurements were conducted with an accelerating voltage of 15 kV, a ~3 µm spot diameter and a beam current of 20 nA. Data were collected with normal counting times of 15–40 s on backgrounds and 20–40 s on peaks (depending on the element considered). In this study, corrections to the Fe^3+^/∑Fe calculated by stoichiometry were small and ranged from 0 to −0.02 (i.e., overestimated value). The error on the corrected Fe^3+^/∑Fe in spinel is ± 0.025. The corrected Fe^3+^/∑Fe in sample spinel are displayed in Supplementary Tables 5 and 6, whereas standard analyses during EPMA runs are given in Supplementary Table 7.

### Sulfur isotope measurements

The S isotope compositions (δ^34^S_measured_, see below for usage of delta notation) of the MP were determined by SIMS using the Cameca IMS 1280 (mono-collector) of the Northeast National Ion Microprobe Facility (NENIMF) at Woods Hole Oceanographic Institution (WHOI; USA). The measurements were conducted using a 10 μm Cs^+^ primary beam, an accelerating voltage of 10 kV and a beam current of 1–2 nA. The secondary ions were collected at an accelerating voltage of 10 kV, with a 150 μm field of view and a mass resolution power (MRP; *M*/∆*M*) of 4000–5500, using an electron multiplier. The energy slit was centred and opened to 40–60 V. Each measurement consisted of fifty cycles for ^32^S and ^34^S, respectively. By operating the Cameca IMS 1280 at a MRP of 5500, it becomes possible to avoid interferences from ^31^P^1^H with ^32^S (ref. ^64^). With this analytical setup, in situ δ^34^S measurements on a 15 × 15 μm area can be conducted with a precision of 0.4–0.6‰ in silicate glasses containing about 500–1600 p.p.m. S (ref. ^64^), i.e., overlapping the range measured in the MP in this study (Supplementary Tables 1 and 11).

The SIMS analysis is based on the measurement of S-bearing glass standards with known S isotope composition to account for the instrumental mass fractionation (IMF)^65^. In-house glass standards at WHOI with major element compositions ranging from basaltic to SiO_2_-rich glasses were used for this calibration^65^. The S isotope composition of these glass standards (δ^34^S_true_) covers a range from about −5.3‰ up to about + 12‰ and was typically determined by KIBA reagent extraction method or calculated on the basis of the isotope composition of the source of S used for the glass syntheses. The nearly 1:1 linear correlation between the KIBA and SIMS results reported earlier^65^ confirms the high analytical accuracy of the independent determination of δ^34^S_true_.

The *y* axis intercept of the linear correlation between KIBA and SIMS results reflects the IMF, which was typically ranging between 0.9935 and 0.995 during our analytical session (Supplementary Table 11). The nearly 1:1 correlation also indicates that the IMF is largely independent of matrix effects related to bulk glass compositions (i.e., major elements and δ^34^S) within the ranges defined by the standards^65^. In addition, variations in the S valence state in silicate glasses are not expected to produce a detectable IMF. This is because the energy involved in the sputtered ion formation process (10–12 keV) completely overwhelms the bond energies in silicate glasses, as was demonstrated by δ^34^S recently determined on experimental glasses equilibrated at variable *f*O_2_ (ref. ^65^).

Correction and filtering of the raw SIMS data (to give (^34^S/^32^S)_corrected_) were realised to account for the variations of IMF during a 50-cycles measurement. In order to monitor short- and long-term variations of IMF and to allow a correction of the raw δ^34^S, at least two measurements on a selected standard (JDF 46°N basaltic glass) were conducted every 2–3 unknown samples (Supplementary Table 11). The δ^34^S_measured_ were then calculated relative to the Vienna Canyon Diablo Troilite (V-CDT) isotope reference standard as follows:

5 $${\mathrm{\delta }}^{{\mathrm{34}}}{\mathrm{S}}_{{\mathrm{measured}}} = \left( {\left( {\,^{34}{\mathrm{S/}}\,^{32}{\mathrm{S}}} \right)_{{\mathrm{corrected}}}{\mathrm{/IMF/0}}{\mathrm{.04416375 - 1}}} \right) \times {\mathrm{1000}}$$

where 0.04416375 is the ^34^S/^32^S for V-CDT having δ^34^S = 0‰. The 2σ errors associated with the δ^34^S_measured_ in this study (0.6–1.6‰, Supplementary Table 11) were propagated from the signal count statistics and the uncertainty in the regression of the SIMS calibration curve. Recent replicate analyses of a MORB glass standard (892-1) have revealed an external reproducibility of SIMS analysis of ± 0.52‰ (*n* = 13, 2*σ*)^65^. More detail on SIMS calibration materials for in situ S isotope analyses of silicate glasses and the processing procedures of raw SIMS data can be found in Fiege et al.^65^.

## Electronic supplementary material

Supplementary Information

## Data Availability

The authors declare that the data generated or analysed during this study are included in this published article and its Supplementary Information files.
