# Supplementary material for: Oxidising agents in sub-arc mantle melts link slab devolatilisation and arc magmas
Source: Nat Commun. 2018 Aug 29;9:3500. doi: 10.1038/s41467-018-05804-2 (PMC6115406; doi:10.1038/s41467-018-05804-2)
Supplement: Supplementary file 1 — Supplementary Information [file 41467_2018_5804_MOESM1_ESM.pdf]

Oxidising agents in sub-arc mantle melts link slab devolatilisation and arc magmas

Supplementary Information

Antoine Bénard *et al.*

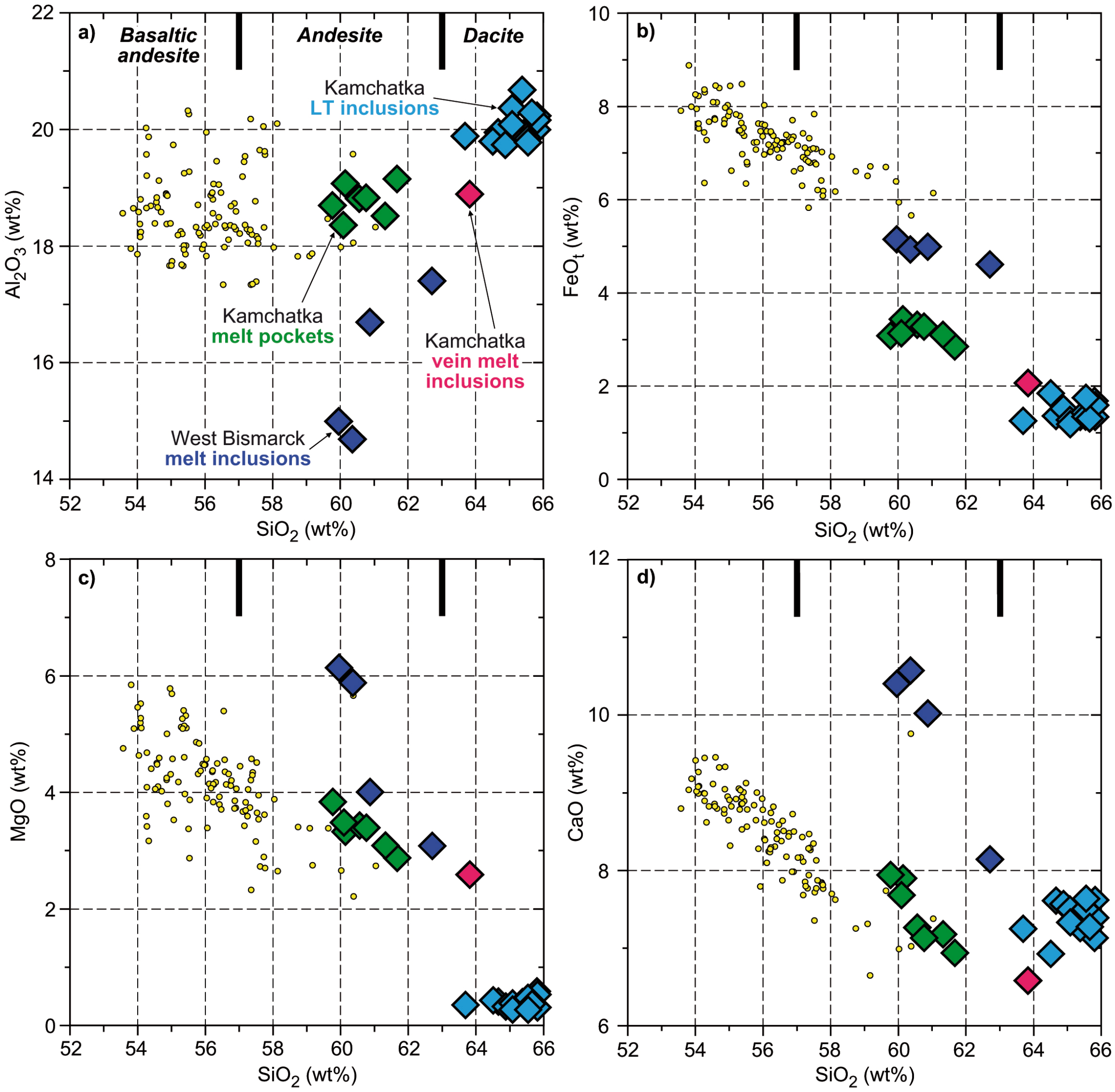

Supplementary Figure 1 | (a–d) Major element compositions (re-calculated on an anhydrous basis) of sub-arc mantle inclusions (EPMA data). The magmas carrying Kamchatka xenoliths[3] (yellow dots) are compared to LT inclusions (cyan diamond), MP (green diamonds) and vein MI (pink diamond) from Kamchatka and West Bismarck MI (blue diamonds) investigated for their S valence state in this study. The magmas carrying West Bismarck xenoliths are picrites[2], which major element compositions range beyond the scale in these plots. Note that the inclusions in these plots are all unheated and free of daughter silicate minerals (*i.e.* they only contain glass as a silicate phase).

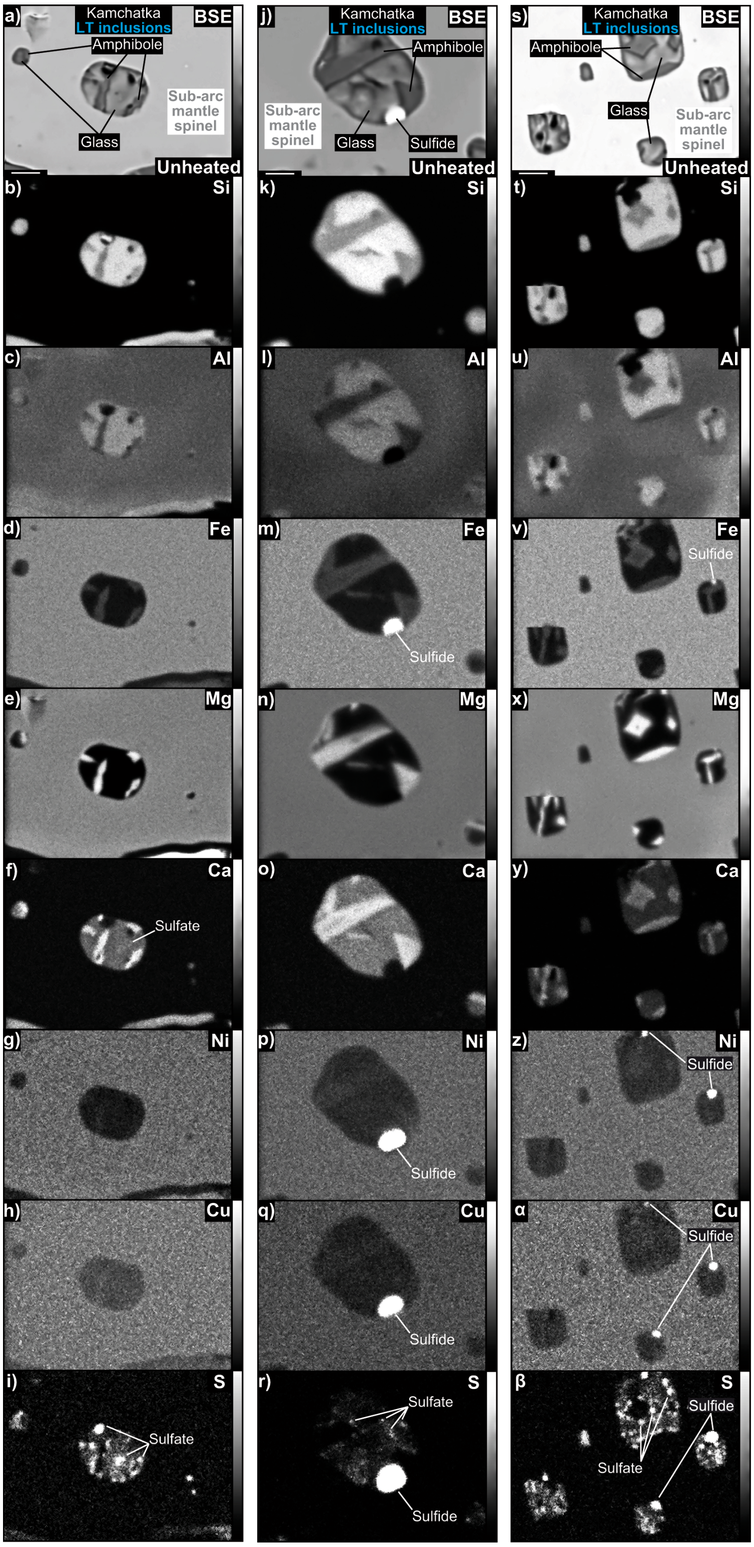

Supplementary Figure 2 | (a, j, s) Back-scattered electron (BSE) images and (b–i, k–r, t–β) chemical maps for sub-arc mantle spinel-hosted LT inclusions from Kamchatka (EPMA data). Scale bar length is ~10 μm. Note that all the inclusions for which data are presented here are unheated.

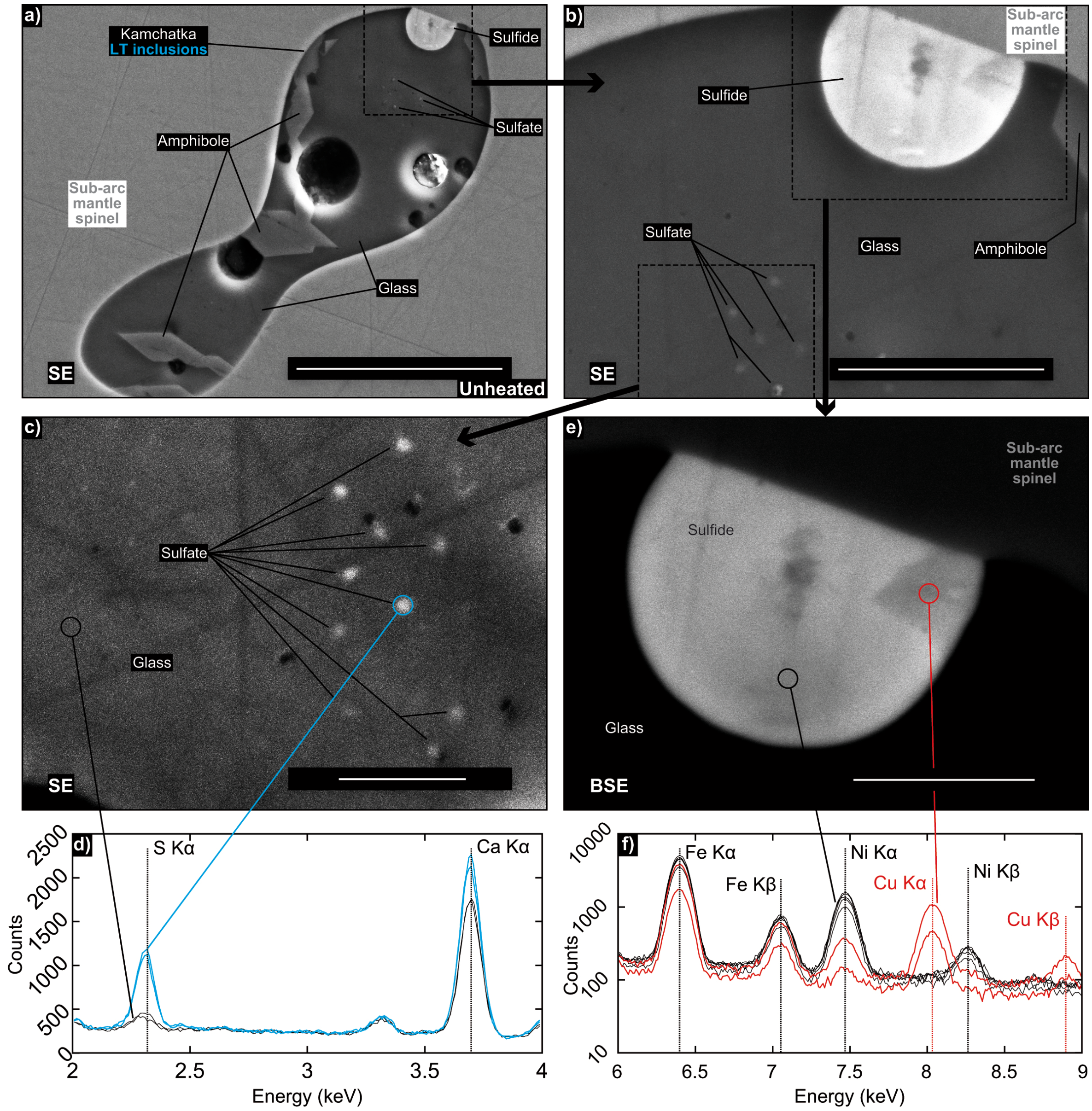

Supplementary Figure 3 | (a–c) Secondary electron (SE) and (e) back-scattered electron (BSE) images with (d, f) EDS spectra for sub-arc mantle spinel-hosted LT inclusions from Kamchatka (SEM data). Scale bar lengths are ~20, ~5, ~2 and ~2.5 µm, respectively in (a), (b), (c) and (e). Note that all the inclusions for which data are presented here are unheated.

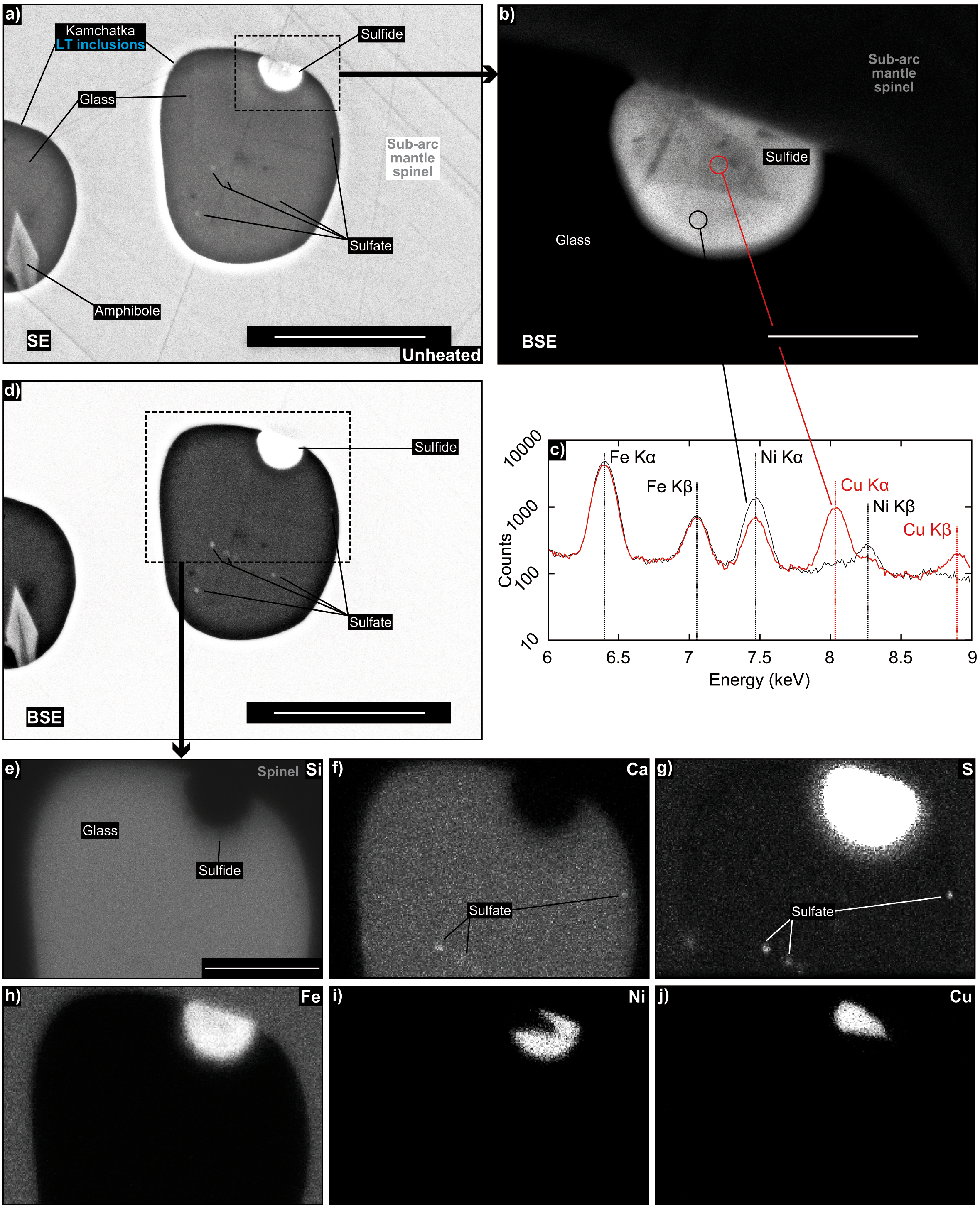

Supplementary Figure 4 | (a, b) Secondary electron (SE) and (d) back-scattered electron (BSE) images, (c) EDS spectra and (e–j) chemical maps for sub-arc mantle spinel-hosted LT inclusions from Kamchatka (SEM data). Scale bar lengths are ~10, ~5, ~10 and ~5 µm, respectively in (a), (b), (d) and (e). Note that all the inclusions for which data are presented here are unheated.

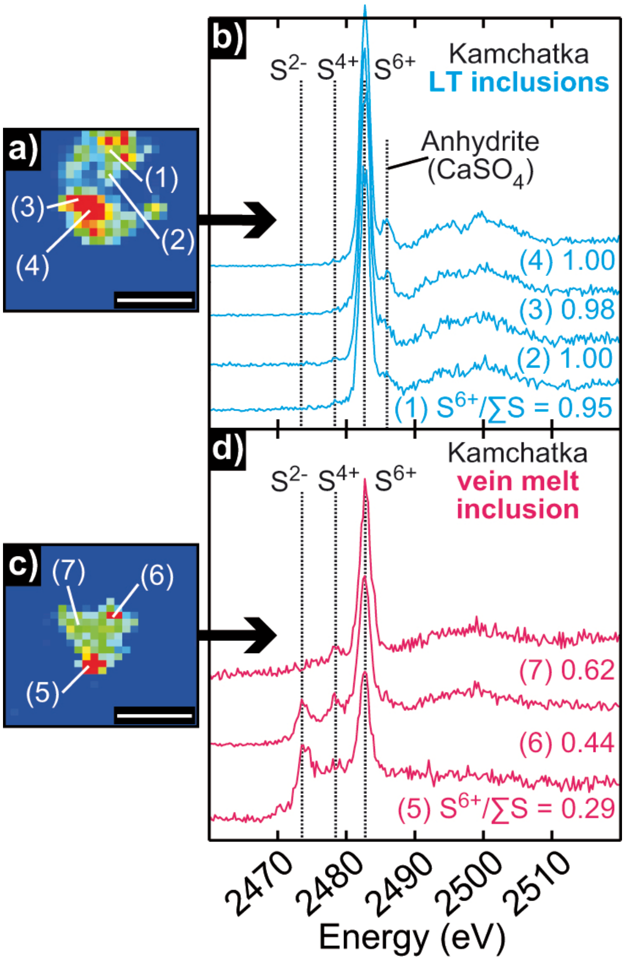

Supplementary Figure 5 l Supplementary sulfur distribution and valence state data for sub-arc mantle spinel-hosted glass (formerly melt) inclusions from Kamchatka. (a) Uncalibrated S Kα X-ray fluorescence maps of an LT inclusion and its (b) S K-edge XANES spectra. (c) Uncalibrated S Kα X-ray fluorescence maps of a vein MI and its (d) S K-edge XANES spectra. Colours in (a, c) range from blue (low S concentrations) to red (high S concentrations). The S-free host spinel appears as dark blue. Numbers refer to individual XANES spot analysis. Spectra numbers refer to the spot position as shown in (a, c). Vertical black lines indicate energies for specific S valence states: 2482.8 eV for S^{6+}, 2478 eV for S^{4+} and 2472.5 eV for S^{2-} in crystalline Fe-S compounds. The additional feature at ~2486 eV is typical for crystalline anhydrite. Some S^{4+} is generated during XANES measurements of glasses by photo-reduction of S^{6+}. Scale bar lengths are ~10 and ~20 µm, respectively in (a) and (c). Note that the inclusions for which data are presented here are unheated.

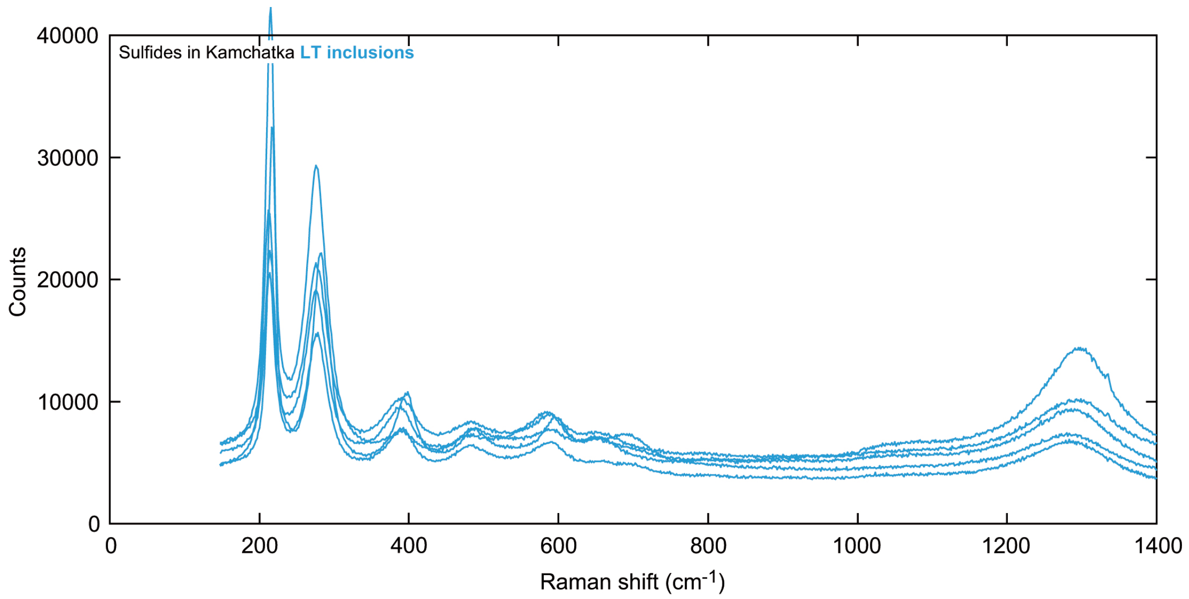

Supplementary Figure 6 | Raman spectra for sub-arc mantle spinel-hosted sulfides in LT inclusions from Kamchatka. Note that all the inclusions for which data are presented here are unheated.

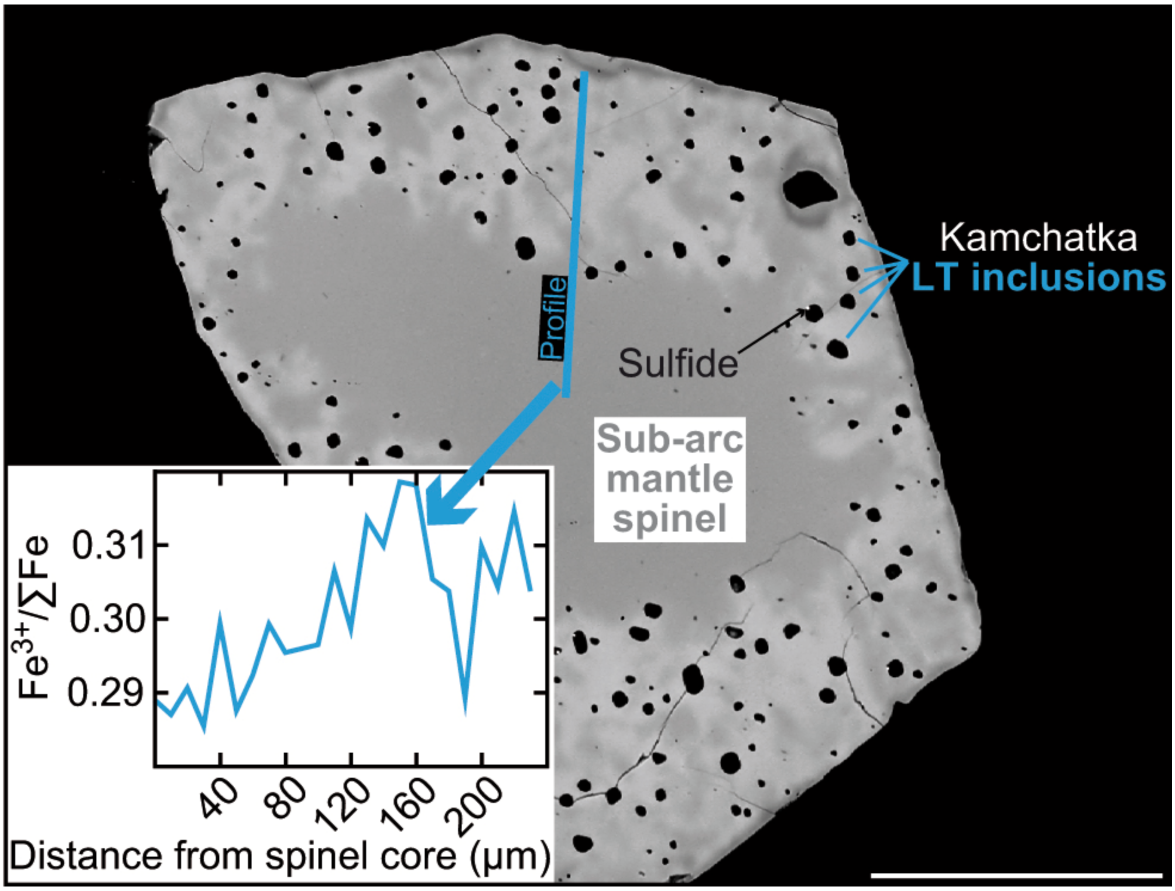

Supplementary Figure 7 | Back-scattered electron (BSE) image indicating the location of a core to rim $Fe^{3+}/\sum Fe$ profile in a sub-arc mantle spinel from Kamchatka with LT inclusions distributed at its rims (EPMA data). Scale bar length is ~200 µm.

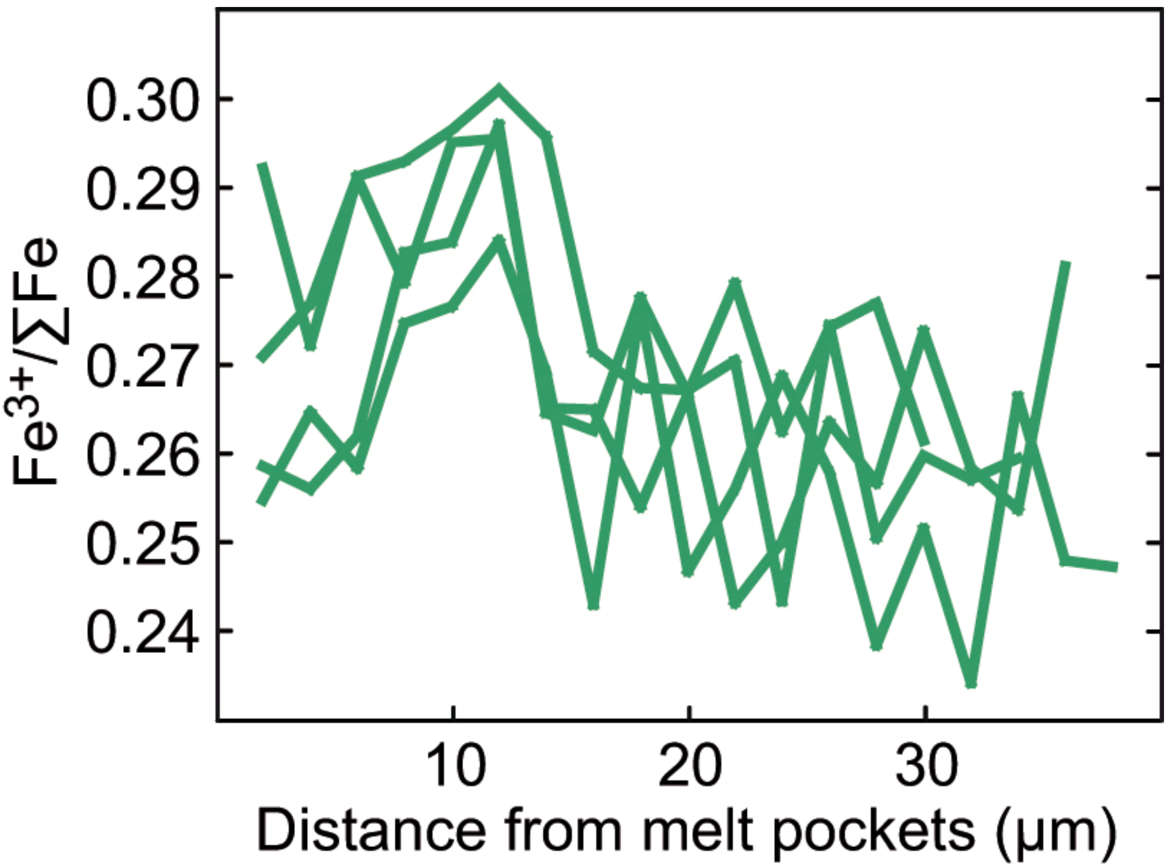

Supplementary Figure 8 | Calculated Fe^{3+}/∑Fe by stoichiometry for profiles adjacent to MP in sub-arc mantle spinel from Kamchatka (data from Ionov *et al.*[3]).

Supplementary Table 1 | Major element (wt%) and sulfur (ppm) abundances and valence state in 'bulk' (*i.e.* potentially containing anhydrite) low-temperature (LT) inclusions, melt pockets (MP) and vein melt inclusions (vein MI) in the Kamchatka sub-arc mantle peridotites in this study

Sample	SiO_2	TiO_2	Al_2O_3	Cr_2O_3	FeO_t[a]	MnO	MgO	CaO	Na_2O	K_2O	P_2O_5	S	Total	Mg#[b]	Fe/S[c]	$S^{6+}/\sum S$[d]
LT-1 (Av33)	57.81	b.d.	17.82	0.48	1.20	b.d.	0.35	6.79	3.57	0.42	0.32	3073	89.38	0.34	1.75	0.98–1.00[e]
LT-2 (Av33)	*57.75*	*0.05*	*17.70*	*0.56*	*1.64*	*b.d.*	*0.37*	*6.19*	*3.60*	*0.57*	*0.11*	*4799*	*89.50*	*0.29*	*1.52*	*1.00*[e]
LT-3 (Av33)	57.83	0.20	17.58	0.52	1.34	0.03	0.28	6.74	3.21	0.58	0.11	3454	89.12	0.27	1.73	0.95–1.00[f]
LT-4 (Av33)	57.18	b.d.	17.83	0.83	1.11	0.13	0.30	6.50	4.17	0.55	b.d.	5771	89.75	0.33	0.86	0.98–1.00[f]
LT-5 (Av33)	*59.00*	*0.08*	*17.91*	*0.65*	*1.49*	*0.10*	*0.51*	*6.38*	*2.21*	*0.58*	*0.11*	*3076*	*89.63*	*0.38*	*2.17*	–
LT-6 (Av33)	*58.17*	*b.d.*	*17.08*	*0.52*	*1.33*	*0.09*	*0.43*	*6.19*	*2.79*	*0.42*	*b.d.*	*3081*	*87.63*	*0.37*	*1.93*	–
LT-7 (Av33)	*58.05*	*0.28*	*17.83*	*0.57*	*1.17*	*0.00*	*0.46*	*6.71*	*1.94*	*0.51*	*b.d.*	*3266*	*88.18*	*0.41*	*1.60*	–
LT-8 (Av33)	*56.99*	*0.08*	*17.82*	*0.56*	*1.10*	*0.04*	*0.32*	*6.56*	*2.97*	*0.54*	*b.d.*	*2887*	*87.56*	*0.34*	*1.71*	–
LT-9 (Av33)	*58.63*	*b.d.*	*18.53*	*0.62*	*1.19*	*b.d.*	*0.35*	*6.50*	*2.41*	*0.54*	*0.32*	*2887*	*89.66*	*0.34*	*1.84*	–
LT-10 (Av33)	*58.02*	*0.08*	*17.76*	*0.64*	*1.39*	*b.d.*	*0.26*	*6.51*	*2.60*	*0.50*	*b.d.*	*1925*	*88.14*	*0.25*	*3.21*	–
LT-11 (Av33)	*57.15*	*0.15*	*17.62*	*0.80*	*1.03*	*0.06*	*0.23*	*6.43*	*3.85*	*0.47*	–	–	*87.80*	*0.28*	–	–
LT-12 (Av33)	*57.35*	*b.d.*	*17.35*	*0.63*	*1.17*	*b.d.*	*0.40*	*6.56*	*3.51*	*0.52*	–	–	*87.48*	*0.38*	–	–
LT-13 (Av33)	*55.88*	*b.d.*	*17.25*	*0.80*	*1.10*	*0.05*	*0.31*	*6.18*	*2.94*	*0.57*	–	–	*85.08*	*0.33*	–	–
LT-14 (Av33)	*57.72*	*0.06*	*17.40*	*0.62*	*1.53*	*b.d.*	*0.23*	*6.71*	*3.24*	*0.53*	–	–	*88.04*	*0.21*	–	–
MP-1 (Av33)	59.72	0.47	18.53	0.28	3.25	b.d.	3.37	7.15	4.50	0.65	0.26	1908	98.57	0.65	7.61	–
MP-2 (Av33)	60.16	0.72	18.80	0.31	3.09	0.08	3.85	7.99	4.31	0.76	0.21	1713	100.63	0.69	8.05	–
MP-3 (Av33)	60.28	0.71	18.40	0.89	3.13	0.09	3.48	7.70	4.49	0.54	0.32	1333	100.28	0.66	10.5	–
MP-4 (Av33)	*60.73*	*0.40*	*19.24*	*0.39*	*3.45*	*0.03*	*3.34*	*7.97*	*4.23*	*0.68*	*0.26*	*1147*	*100.95*	*0.63*	*13.4*	*0.55–0.58*[e]
MP-5 (Av33)	60.44	0.37	18.71	0.88	3.25	0.01	3.36	7.08	4.28	0.73	0.11	1145	99.44	0.65	12.7	–
MP-6 (Av33)	60.40	0.17	18.22	0.37	3.04	0.07	3.03	7.06	4.67	0.74	0.42	1338	98.46	0.64	10.1	–
MP-7 (Av33)	60.65	0.42	18.82	0.44	2.79	0.01	2.82	6.81	4.61	0.70	0.11	764	98.32	0.64	16.3	–
Vein MI-1 (Av25)	62.48	0.03	18.48	n.d.	2.00	0.09	2.53	6.43	3.61	1.06	n.d.	155	97.86	0.69	57.6	0.24–0.88[e]
Vein MI-2 (Av24)	65.11	b.d.	16.62	n.d.	0.37	0.05	0.18	4.27	3.61	0.25	n.d.	345	91.93	0.47	4.83	0.29–0.62[f]

Major element analyses by EPMA (shown in Supplementary Fig. 1)

Analyses in italics are those of inclusions also characterised by Raman spectrometry (shown in Figs 2h, i & 3c)

The major element and S data for vein MI are from Bénard *et al.*[5]

b.d., below detection

[a]All Fe treated as Fe^{2+}

[b]$Mg/(Mg+Fe_t)$

[c]Molar ratio

[d]$\sum S=S^{2-}+S^{6+}$ (XANES data)

[e]Shown in Fig. 2b–g

[f]Shown in Supplementary Fig. 5

Supplementary Table 2 | Major element and sulfur abundances (wt%) in 'pure' glasses (*i.e.* free of anhydrite) of low-temperature (LT) inclusions in the Kamchatka sub-arc mantle peridotite in this study

Sample	Si	Ti	Al	Cr	Fe_t[a]	Ca	Na	K	S	O	Total	Mg#[b]	Fe/S[c]
LT-15 (Av33)	26.45	0.09	9.17	0.54	0.90	3.95	0.39	0.37	0.29	41.07	83.22	–	1.79
LT-16 (Av33)	26.05	b.d.	9.01	0.48	0.70	3.85	0.67	0.42	0.18	40.24	81.60	–	2.23

Major element analyses by SEM (shown in Supplementary Figs 3 & 4)
b.d., below detection
[a]All Fe treated as Fe^{2+}
[b]$Mg/(Mg+Fe_t)$
[c]Molar ratio

Supplementary Table 3 | Major element (wt%) and sulfur (ppm) abundances in 'bulk' (*i.e.* potentially containing anhydrite) melt inclusions (MI) in the West Bismarck sub-arc mantle peridotite in this study

Sample	SiO_2	TiO_2	Al_2O_3	Cr_2O_3	FeO_t[a]	MnO	MgO	CaO	Na_2O	K_2O	NiO	S	Total	Mg#[b]	Fe/S[c]
MI-1 (67-02D(7))	*58.72*	*0.46*	*14.67*	*0.79*	*5.03*	*0.10*	*6.00*	*10.18*	*1.43*	*0.53*	*b.d.*	*1397*	*97.92*	*0.68*	*16.1*
MI-2 (67-02D(7))	*59.02*	*0.58*	*14.35*	*0.86*	*4.81*	*0.10*	*5.73*	*10.33*	*1.41*	*0.56*	*0.01*	*1745*	*97.76*	*0.68*	*12.3*
MI-3 (67-02D(7))	*62.46*	*0.60*	*17.32*	*b.d.*	*4.57*	*0.08*	*3.06*	*8.10*	*2.44*	*0.95*	*b.d.*	*2510*	*99.58*	*0.54*	*8.13*
MI-4 (67-02D(7))	*60.73*	*0.52*	*16.64*	*0.05*	*4.96*	*0.10*	*3.98*	*9.99*	*1.92*	*0.83*	*0.02*	*1119*	*99.74*	*0.59*	*19.8*

Major element analyses by EPMA (shown in Supplementary Fig. 1)
Analyses in italics are those of inclusions also characterised by Raman spectrometry (shown in Figs 2h, i & 3c)
b.d., below detection
[a]all Fe treated as Fe^{2+}
[b]$Mg/(Mg+Fe_t)$
[c]Molar ratio

Supplementary Table 4 | Major element abundances (wt%) in sulfides of low-temperature (LT) inclusions in the Kamchatka sub-arc mantle peridotite in this study

Sample	S	Fe	Ni	Cu	Total
LT-15 (Av33)	*40.7*	*37.6*	*21.7*	*b.d.*	*97.9*
LT-15 (Av33)	*34.6*	*36.8*	*4.5*	*24.1*	*96.4*
LT-16 (Av33)	*39.7*	*41.2*	*19.1*	*b.d.*	*97.8*
LT-16 (Av33)	*37.1*	*35.8*	*8.5*	*18.6*	*96.8*
LT-17 (Av33)	*40.2*	*40.3*	*18.7*	*0.8*	*97.8*
LT-18 (Av33)	40.4	38.7	20.5	0.4	97.8
LT-19 (Av33)	40.3	41.3	18.4	b.d.	97.9

Major element analyses by SEM (shown in Supplementary Figs 3 & 4)

Analyses in italics are those of sulfides also characterised by Raman spectrometry (shown in Supplementary Fig. 6)

b.d., below detection

Supplementary Table 5 | Major element abundances (At%) in spinel near low-temperature (LT) inclusions in the Kamchatka sub-arc mantle peridotite in this study

Sample	Analysis	$Fe^{3+}_{corrected}$	Cr	$Fe^{2+}_{corrected}$	Al	Mg	$\sum Fe_{corrected}$[a]	$Fe^{3+}/\sum Fe_{corrected}$	Mg#[b]	Cr#[c]	Distance[d] (μm)
**Profile 1 (Raman spectrum for the corresponding inclusion is shown in Fig. 3c)**											
LT-2 (Av33)	av33sp1prof2	0.18	1.15	0.35	0.66	0.65	0.52	0.34	0.55	0.64	9
LT-2 (Av33)	av33sp1prof2	0.17	1.16	0.35	0.66	0.65	0.52	0.33	0.55	0.64	12
LT-2 (Av33)	av33sp1prof2	0.17	1.18	0.35	0.64	0.64	0.52	0.32	0.55	0.65	15
LT-2 (Av33)	av33sp1prof2	0.17	1.19	0.35	0.63	0.65	0.52	0.33	0.55	0.65	18
LT-2 (Av33)	av33sp1prof2	0.17	1.18	0.35	0.64	0.65	0.52	0.32	0.55	0.65	21
LT-2 (Av33)	av33sp1prof2	0.17	1.16	0.35	0.66	0.65	0.52	0.33	0.56	0.64	24
LT-2 (Av33)	av33sp1prof2	0.17	1.15	0.35	0.67	0.65	0.52	0.33	0.56	0.63	27
LT-2 (Av33)	av33sp1prof2	0.17	1.15	0.35	0.66	0.65	0.52	0.33	0.56	0.64	30
LT-2 (Av33)	av33sp1prof2	0.17	1.17	0.35	0.65	0.65	0.52	0.33	0.56	0.64	33
LT-2 (Av33)	av33sp1prof2	0.17	1.19	0.35	0.63	0.64	0.52	0.32	0.55	0.65	36
LT-2 (Av33)	av33sp1prof2	0.17	1.22	0.35	0.60	0.65	0.52	0.32	0.55	0.67	39
LT-2 (Av33)	av33sp1prof2	0.16	1.23	0.35	0.59	0.64	0.52	0.32	0.55	0.68	42
LT-2 (Av33)	av33sp1prof2	0.16	1.24	0.36	0.59	0.64	0.52	0.31	0.55	0.68	45
LT-2 (Av33)	av33sp1prof2	0.16	1.25	0.36	0.58	0.63	0.52	0.30	0.55	0.68	48
LT-2 (Av33)	av33sp1prof2	0.16	1.25	0.36	0.58	0.64	0.52	0.31	0.55	0.68	51
LT-2 (Av33)	av33sp1prof2	0.16	1.26	0.37	0.57	0.63	0.52	0.30	0.55	0.69	54
LT-2 (Av33)	av33sp1prof2	0.16	1.27	0.36	0.56	0.64	0.52	0.30	0.55	0.69	57
**Profile 2 (Raman spectrum for the corresponding inclusion is shown in Fig. 2h)**											
LT-5 (Av33)	av33sp1prof4	0.16	1.17	0.36	0.64	0.65	0.52	0.31	0.56	0.65	6
LT-5 (Av33)	av33sp1prof4	0.16	1.19	0.35	0.63	0.65	0.51	0.32	0.56	0.65	8
LT-5 (Av33)	av33sp1prof4	0.16	1.20	0.36	0.63	0.64	0.51	0.30	0.56	0.65	10
LT-5 (Av33)	av33sp1prof4	0.16	1.20	0.35	0.63	0.64	0.51	0.31	0.56	0.66	12
LT-5 (Av33)	av33sp1prof4	0.16	1.21	0.36	0.62	0.64	0.52	0.31	0.55	0.66	14
LT-5 (Av33)	av33sp1prof4	0.16	1.22	0.36	0.61	0.64	0.51	0.31	0.55	0.67	16
LT-5 (Av33)	av33sp1prof4	0.16	1.23	0.36	0.60	0.64	0.51	0.30	0.56	0.67	18
LT-5 (Av33)	av33sp1prof4	0.16	1.22	0.36	0.61	0.64	0.51	0.30	0.56	0.67	20
LT-5 (Av33)	av33sp1prof4	0.15	1.21	0.36	0.61	0.64	0.51	0.30	0.56	0.66	22
LT-5 (Av33)	av33sp1prof4	0.15	1.21	0.36	0.61	0.64	0.51	0.30	0.56	0.66	24
LT-5 (Av33)	av33sp1prof4	0.15	1.20	0.36	0.63	0.64	0.51	0.30	0.55	0.66	26
LT-5 (Av33)	av33sp1prof4	0.16	1.20	0.36	0.62	0.64	0.52	0.30	0.55	0.66	28
LT-5 (Av33)	av33sp1prof4	0.16	1.20	0.35	0.62	0.65	0.52	0.31	0.56	0.66	30
LT-5 (Av33)	av33sp1prof4	0.16	1.20	0.36	0.62	0.64	0.51	0.31	0.56	0.66	32

LT-5 (Av33)	av33sp1prof4	0.16	1.20	0.35	0.61	0.65	0.51	0.31	0.56	0.66	34
LT-5 (Av33)	av33sp1prof4	0.15	1.20	0.36	0.62	0.64	0.51	0.30	0.56	0.66	36
LT-5 (Av33)	av33sp1prof4	0.15	1.17	0.35	0.65	0.65	0.51	0.30	0.56	0.64	38
**Profile 3 (from core to rim of a grain rimmed with LT inclusions; Supplementary Fig. 7)**											
Av33	av33sp3prof1	0.14	1.19	0.35	0.66	0.65	0.49	0.29	0.57	0.64	0
Av33	av33sp3prof1	0.14	1.20	0.34	0.66	0.65	0.48	0.29	0.57	0.64	10
Av33	av33sp3prof1	0.14	1.19	0.35	0.66	0.65	0.49	0.29	0.57	0.64	20
Av33	av33sp3prof1	0.14	1.19	0.35	0.66	0.64	0.49	0.29	0.57	0.64	30
Av33	av33sp3prof1	0.15	1.19	0.34	0.66	0.65	0.49	0.30	0.57	0.64	40
Av33	av33sp3prof1	0.14	1.19	0.35	0.66	0.65	0.49	0.29	0.57	0.64	50
Av33	av33sp3prof1	0.14	1.19	0.35	0.66	0.65	0.50	0.29	0.57	0.64	60
Av33	av33sp3prof1	0.15	1.19	0.35	0.65	0.65	0.49	0.30	0.57	0.65	70
Av33	av33sp3prof1	0.15	1.19	0.35	0.66	0.64	0.50	0.30	0.56	0.64	80
Av33	av33sp3prof1	0.15	1.24	0.36	0.60	0.64	0.51	0.30	0.56	0.67	100
Av33	av33sp3prof1	0.16	1.24	0.36	0.60	0.64	0.51	0.31	0.55	0.67	110
Av33	av33sp3prof1	0.15	1.27	0.36	0.57	0.63	0.51	0.30	0.55	0.69	120
Av33	av33sp3prof1	0.16	1.23	0.36	0.60	0.64	0.52	0.31	0.55	0.67	130
Av33	av33sp3prof1	0.16	1.23	0.36	0.61	0.64	0.52	0.31	0.55	0.67	140
Av33	av33sp3prof1	0.17	1.22	0.36	0.60	0.64	0.52	0.32	0.55	0.67	150
Av33	av33sp3prof1	0.17	1.23	0.36	0.60	0.64	0.53	0.32	0.55	0.67	160
Av33	av33sp3prof1	0.16	1.25	0.37	0.58	0.63	0.53	0.31	0.54	0.68	170
Av33	av33sp3prof1	0.16	1.25	0.37	0.59	0.63	0.53	0.30	0.54	0.68	180
Av33	av33sp3prof1	0.15	1.23	0.37	0.59	0.63	0.53	0.29	0.55	0.68	190
Av33	av33sp3prof1	0.16	1.25	0.36	0.58	0.63	0.53	0.31	0.54	0.69	200
Av33	av33sp3prof1	0.16	1.25	0.36	0.58	0.63	0.52	0.30	0.55	0.68	210
Av33	av33sp3prof1	0.16	1.20	0.36	0.63	0.64	0.52	0.31	0.55	0.66	220
Av33	av33sp3prof1	0.16	1.22	0.36	0.61	0.64	0.51	0.30	0.55	0.67	230
**Profile 4 (Raman spectrum for the corresponding inclusion is shown in Fig. 2h)**											
LT-6 (Av33)	av33sp1prof1	0.16	1.14	0.34	0.68	0.65	0.50	0.32	0.56	0.63	6
LT-6 (Av33)	av33sp1prof1	0.16	1.16	0.35	0.67	0.65	0.51	0.32	0.56	0.63	9
LT-6 (Av33)	av33sp1prof1	0.16	1.19	0.35	0.63	0.65	0.51	0.31	0.56	0.65	12
LT-6 (Av33)	av33sp1prof1	0.15	1.23	0.36	0.60	0.64	0.51	0.30	0.56	0.67	15
LT-6 (Av33)	av33sp1prof1	0.15	1.26	0.36	0.58	0.64	0.51	0.29	0.56	0.69	18
LT-6 (Av33)	av33sp1prof1	0.15	1.26	0.36	0.58	0.64	0.51	0.30	0.55	0.69	21
LT-6 (Av33)	av33sp1prof1	0.15	1.25	0.36	0.58	0.64	0.51	0.30	0.56	0.68	24
LT-6 (Av33)	av33sp1prof1	0.15	1.24	0.36	0.59	0.64	0.51	0.30	0.56	0.68	33

LT-6 (Av33)	av33sp1prof1	0.15	1.24	0.36	0.59	0.64	0.51	0.30	0.56	0.68	36
LT-6 (Av33)	av33sp1prof1	0.15	1.24	0.36	0.59	0.64	0.51	0.30	0.55	0.68	39
LT-6 (Av33)	av33sp1prof1	0.15	1.24	0.36	0.59	0.64	0.51	0.30	0.56	0.68	42
LT-6 (Av33)	av33sp1prof1	0.16	1.25	0.36	0.58	0.64	0.52	0.31	0.55	0.68	45
LT-6 (Av33)	av33sp1prof1	0.16	1.26	0.36	0.57	0.64	0.52	0.30	0.55	0.69	48
LT-6 (Av33)	av33sp1prof1	0.16	1.25	0.36	0.58	0.64	0.52	0.30	0.55	0.68	51
LT-6 (Av33)	av33sp1prof1	0.16	1.25	0.36	0.58	0.64	0.51	0.31	0.56	0.68	54
LT-6 (Av33)	av33sp1prof1	0.16	1.25	0.36	0.57	0.64	0.52	0.31	0.55	0.69	57
**Profile 5 (Raman spectrum for the corresponding inclusion is shown in Fig. 2h)**											
LT-7 (Av33)	av33sp1prof3	0.15	1.23	0.36	0.60	0.64	0.51	0.30	0.55	0.67	6
LT-7 (Av33)	av33sp1prof3	0.16	1.21	0.36	0.62	0.64	0.52	0.31	0.55	0.66	9
LT-7 (Av33)	av33sp1prof3	0.17	1.16	0.35	0.65	0.65	0.52	0.32	0.56	0.64	12
LT-7 (Av33)	av33sp1prof3	0.17	1.15	0.35	0.66	0.65	0.52	0.33	0.56	0.63	15
LT-7 (Av33)	av33sp1prof3	0.17	1.16	0.35	0.66	0.65	0.52	0.33	0.56	0.64	18
LT-7 (Av33)	av33sp1prof3	0.17	1.18	0.36	0.64	0.64	0.52	0.32	0.55	0.65	21
LT-7 (Av33)	av33sp1prof3	0.17	1.19	0.36	0.62	0.64	0.52	0.32	0.55	0.66	24
LT-7 (Av33)	av33sp1prof3	0.17	1.20	0.36	0.62	0.64	0.52	0.32	0.55	0.66	27
LT-7 (Av33)	av33sp1prof3	0.16	1.20	0.36	0.62	0.64	0.52	0.31	0.55	0.66	30
LT-7 (Av33)	av33sp1prof3	0.16	1.21	0.36	0.61	0.64	0.52	0.31	0.55	0.66	33
LT-7 (Av33)	av33sp1prof3	0.17	1.20	0.35	0.62	0.64	0.52	0.32	0.55	0.66	36
LT-7 (Av33)	av33sp1prof3	0.17	1.17	0.35	0.65	0.65	0.52	0.33	0.55	0.64	39
LT-7 (Av33)	av33sp1prof3	0.17	1.15	0.35	0.66	0.65	0.52	0.33	0.55	0.64	42
LT-7 (Av33)	av33sp1prof3	0.17	1.17	0.36	0.65	0.64	0.52	0.32	0.55	0.64	45
LT-7 (Av33)	av33sp1prof3	0.17	1.20	0.36	0.62	0.64	0.52	0.32	0.55	0.66	48
LT-7 (Av33)	av33sp1prof3	0.17	1.22	0.36	0.60	0.64	0.53	0.32	0.55	0.67	51
LT-7 (Av33)	av33sp1prof3	0.16	1.25	0.37	0.58	0.63	0.53	0.30	0.55	0.68	54
LT-7 (Av33)	av33sp1prof3	0.16	1.26	0.37	0.57	0.63	0.53	0.30	0.55	0.69	57
LT-7 (Av33)	av33sp1prof3	0.16	1.26	0.36	0.56	0.63	0.53	0.31	0.55	0.69	60
LT-7 (Av33)	av33sp1prof3	0.16	1.26	0.37	0.56	0.63	0.53	0.31	0.54	0.69	63
LT-7 (Av33)	av33sp1prof3	0.15	1.27	0.37	0.56	0.63	0.52	0.29	0.55	0.70	66
LT-7 (Av33)	av33sp1prof3	0.16	1.27	0.37	0.56	0.63	0.52	0.30	0.55	0.69	69
**Profile 6 (Raman spectrum for the corresponding inclusion is shown in Fig. 2h)**											
LT-8 (Av33)	av33sp2prof1	0.16	1.19	0.36	0.63	0.64	0.52	0.31	0.55	0.65	4
LT-8 (Av33)	av33sp2prof1	0.16	1.19	0.36	0.63	0.64	0.52	0.31	0.55	0.66	6
LT-8 (Av33)	av33sp2prof1	0.16	1.21	0.36	0.62	0.64	0.52	0.31	0.55	0.66	8
LT-8 (Av33)	av33sp2prof1	0.15	1.23	0.36	0.60	0.64	0.51	0.30	0.55	0.67	10

LT-8 (Av33)	av33sp2prof1	0.16	1.24	0.37	0.59	0.63	0.52	0.30	0.55	0.68	12
LT-8 (Av33)	av33sp2prof1	0.16	1.25	0.36	0.58	0.63	0.52	0.30	0.55	0.68	14
LT-8 (Av33)	av33sp2prof1	0.16	1.24	0.36	0.59	0.64	0.52	0.30	0.55	0.68	16
LT-8 (Av33)	av33sp2prof1	0.15	1.25	0.36	0.58	0.64	0.51	0.29	0.55	0.68	18
LT-8 (Av33)	av33sp2prof1	0.16	1.24	0.36	0.59	0.64	0.52	0.30	0.55	0.68	20
LT-8 (Av33)	av33sp2prof1	0.16	1.24	0.36	0.59	0.64	0.52	0.31	0.55	0.68	22
LT-8 (Av33)	av33sp2prof1	0.15	1.24	0.36	0.59	0.64	0.52	0.30	0.55	0.68	24
LT-8 (Av33)	av33sp2prof1	0.15	1.24	0.36	0.59	0.64	0.51	0.30	0.55	0.68	26
LT-8 (Av33)	av33sp2prof1	0.15	1.23	0.36	0.60	0.64	0.51	0.30	0.55	0.67	28
**Profile 7 (Raman spectrum for the corresponding inclusion is shown in Fig. 2h)**											
LT-9 (Av33)	av33sp2prof2	0.17	1.21	0.36	0.61	0.64	0.53	0.32	0.54	0.67	6
LT-9 (Av33)	av33sp2prof2	0.17	1.21	0.36	0.60	0.64	0.53	0.33	0.55	0.67	8
LT-9 (Av33)	av33sp2prof2	0.17	1.21	0.36	0.60	0.64	0.53	0.32	0.54	0.67	11
LT-9 (Av33)	av33sp2prof2	0.17	1.21	0.36	0.61	0.64	0.53	0.32	0.54	0.67	13
LT-9 (Av33)	av33sp2prof2	0.17	1.21	0.36	0.61	0.64	0.53	0.32	0.55	0.66	15
LT-9 (Av33)	av33sp2prof2	0.17	1.20	0.36	0.61	0.64	0.53	0.32	0.54	0.66	17
LT-9 (Av33)	av33sp2prof2	0.16	1.20	0.36	0.62	0.64	0.52	0.31	0.55	0.66	19
LT-9 (Av33)	av33sp2prof2	0.16	1.20	0.36	0.62	0.63	0.53	0.31	0.55	0.66	21
LT-9 (Av33)	av33sp2prof2	0.17	1.20	0.36	0.62	0.64	0.53	0.32	0.55	0.66	23
LT-9 (Av33)	av33sp2prof2	0.16	1.21	0.36	0.62	0.64	0.53	0.31	0.55	0.66	25
LT-9 (Av33)	av33sp2prof2	0.16	1.20	0.36	0.62	0.64	0.53	0.31	0.55	0.66	27
LT-9 (Av33)	av33sp2prof2	0.17	1.21	0.36	0.61	0.64	0.53	0.31	0.55	0.66	29
LT-9 (Av33)	av33sp2prof2	0.16	1.22	0.36	0.61	0.64	0.52	0.31	0.55	0.67	32
LT-9 (Av33)	av33sp2prof2	0.16	1.23	0.36	0.60	0.63	0.52	0.31	0.55	0.67	34
LT-9 (Av33)	av33sp2prof2	0.16	1.23	0.36	0.60	0.63	0.52	0.30	0.55	0.67	36

Major element analyses by EPMA with spinel secondary standards analysed by Mössbauer (shown in Fig. 3d & Supplementary Fig. 7). Thin horizontal lines separate excluded (high and variable $Fe^{3+}/\sum Fe$ next to melt inclusions) from selected (relatively low and constant $Fe^{3+}/\sum Fe$ far from melt inclusions) data to calculate fO_2 recorded in the Kamchatka sub-arc mantle peridotites in this study; this is the opposite case for the core to rim profile 3 (shown in Supplementary Fig. 7)

[a] $\sum Fe = Fe^{2+} + Fe^{3+}$

[b] $Mg/(Mg+Fe_t)$

[c] Cr/(Cr+Al)

[d] Distance from melt inclusions (in microns)

Supplementary Table 6 | Major element abundances (At%) in spinel near melt inclusions (MI) in the West Bismarck sub-arc mantle peridotite in this study

Sample	Analysis	$Fe^{3+}_{corrected}$	Cr	$Fe^{2+}_{corrected}$	Al	Mg	$\sum Fe_{corrected}$[a]	$Fe^{3+}/\sum Fe_{corrected}$	Mg#[b]	Cr#[c]	Distance[d] (μm)
**Profile 1 (Raman spectrum for the corresponding inclusion is shown in Fig. 3c)**											
MI-1 (67-02D(7))	Line 3 67_02d7sp2prof1	0.16	1.06	0.34	0.75	0.67	0.50	0.32	0.58	0.59	6
MI-1 (67-02D(7))	Line 4 67_02d7sp2prof1	0.15	1.08	0.33	0.75	0.67	0.49	0.32	0.58	0.59	9
MI-1 (67-02D(7))	Line 5 67_02d7sp2prof1	0.15	1.05	0.34	0.78	0.66	0.49	0.31	0.58	0.57	12
MI-1 (67-02D(7))	Line 6 67_02d7sp2prof1	0.15	1.16	0.35	0.68	0.65	0.49	0.30	0.57	0.63	15
MI-1 (67-02D(7))	Line 7 67_02d7sp2prof1	0.15	1.18	0.35	0.66	0.65	0.50	0.30	0.56	0.64	18
MI-1 (67-02D(7))	Line 8 67_02d7sp2prof1	0.15	1.16	0.34	0.68	0.65	0.49	0.30	0.57	0.63	21
MI-1 (67-02D(7))	Line 9 67_02d7sp2prof1	0.15	1.07	0.33	0.76	0.66	0.49	0.32	0.58	0.59	24
MI-1 (67-02D(7))	Line 10 67_02d7sp2prof1	0.15	1.04	0.33	0.80	0.67	0.49	0.32	0.58	0.57	27
MI-1 (67-02D(7))	Line 11 67_02d7sp2prof1	0.14	1.22	0.36	0.63	0.64	0.50	0.28	0.56	0.66	30
MI-1 (67-02D(7))	Line 12 67_02d7sp2prof1	0.14	1.28	0.36	0.58	0.63	0.50	0.27	0.56	0.69	33
MI-1 (67-02D(7))	Line 13 67_02d7sp2prof1	0.14	1.27	0.37	0.59	0.63	0.50	0.27	0.55	0.68	36
MI-1 (67-02D(7))	Line 14 67_02d7sp2prof1	0.14	1.26	0.36	0.59	0.63	0.50	0.28	0.56	0.68	39
MI-1 (67-02D(7))	Line 15 67_02d7sp2prof1	0.14	1.27	0.36	0.58	0.63	0.50	0.28	0.56	0.69	42
MI-1 (67-02D(7))	Line 16 67_02d7sp2prof1	0.14	1.29	0.37	0.57	0.63	0.50	0.27	0.56	0.69	45
MI-1 (67-02D(7))	Line 17 67_02d7sp2prof1	0.14	1.29	0.36	0.56	0.63	0.51	0.28	0.55	0.70	48
MI-1 (67-02D(7))	Line 18 67_02d7sp2prof1	0.14	1.28	0.36	0.56	0.63	0.51	0.28	0.55	0.69	51
MI-1 (67-02D(7))	Line 19 67_02d7sp2prof1	0.14	1.29	0.37	0.56	0.63	0.50	0.27	0.56	0.70	54
MI-1 (67-02D(7))	Line 20 67_02d7sp2prof1	0.14	1.30	0.37	0.56	0.63	0.50	0.27	0.56	0.70	57
MI-1 (67-02D(7))	Line 21 67_02d7sp2prof1	0.14	1.29	0.37	0.57	0.63	0.50	0.27	0.56	0.69	60
MI-1 (67-02D(7))	Line 22 67_02d7sp2prof1	0.14	1.28	0.36	0.57	0.63	0.50	0.28	0.56	0.69	63
MI-1 (67-02D(7))	Line 23 67_02d7sp2prof1	0.14	1.26	0.36	0.59	0.63	0.50	0.27	0.56	0.68	66
MI-1 (67-02D(7))	Line 24 67_02d7sp2prof1	0.14	1.26	0.36	0.59	0.64	0.50	0.28	0.56	0.68	69
**Profile 2 (Raman spectrum for the corresponding inclusion is shown in Fig. 2i)**											
MI-2 (67-02D(7))	67_02d7sp2p2	0.17	1.05	0.34	0.76	0.67	0.50	0.33	0.57	0.58	3
MI-2 (67-02D(7))	67_02d7sp2p2	0.16	1.07	0.34	0.75	0.67	0.50	0.33	0.57	0.59	6
MI-2 (67-02D(7))	67_02d7sp2p2	0.15	1.10	0.34	0.73	0.66	0.49	0.31	0.57	0.60	9
MI-2 (67-02D(7))	67_02d7sp2p2	0.15	1.08	0.34	0.75	0.66	0.49	0.31	0.57	0.59	12
MI-2 (67-02D(7))	67_02d7sp2p2	0.15	1.08	0.34	0.76	0.66	0.49	0.31	0.58	0.59	15
MI-2 (67-02D(7))	67_02d7sp2p2	0.14	1.19	0.35	0.65	0.64	0.50	0.29	0.56	0.65	18

MI-2 (67-02D(7))	67_02d7sp2p2	0.14	1.26	0.36	0.60	0.63	0.50	0.27	0.56	0.68	21
MI-2 (67-02D(7))	67_02d7sp2p2	0.14	1.26	0.36	0.60	0.63	0.50	0.27	0.56	0.68	24
MI-2 (67-02D(7))	67_02d7sp2p2	0.14	1.26	0.36	0.59	0.63	0.50	0.27	0.56	0.68	27
MI-2 (67-02D(7))	67_02d7sp2p2	0.14	1.25	0.36	0.60	0.64	0.50	0.28	0.56	0.68	30
MI-2 (67-02D(7))	67_02d7sp2p2	0.14	1.26	0.36	0.60	0.64	0.50	0.28	0.56	0.68	33
MI-2 (67-02D(7))	67_02d7sp2p2	0.14	1.25	0.36	0.60	0.64	0.50	0.29	0.56	0.68	36
**Spot analyses**											
67-02D(7)	67_02d7sp2p549	0.14	1.26	0.36	0.59	0.63	0.51	0.28	0.56	0.68	–
67-02D(7)	67_02d7sp2p550	0.15	1.29	0.36	0.55	0.63	0.51	0.29	0.56	0.70	–
67-02D(7)	67_02d7sp2p551	0.15	1.25	0.36	0.59	0.63	0.51	0.29	0.55	0.68	–
**Profile 3 (Raman spectrum for the corresponding inclusion is shown in Fig. 2i)**											
MI-3 (67-02D(7))	Line 7 67_02d7sp1prof1	0.17	1.10	0.32	0.72	0.68	0.49	0.35	0.58	0.61	14
MI-3 (67-02D(7))	Line 8 67_02d7sp1prof1	0.17	1.11	0.33	0.71	0.67	0.50	0.34	0.58	0.61	16
MI-3 (67-02D(7))	Line 9 67_02d7sp1prof1	0.16	1.12	0.33	0.71	0.67	0.49	0.33	0.58	0.61	18
MI-3 (67-02D(7))	Line 10 67_02d7sp1prof1	0.16	1.12	0.33	0.71	0.67	0.49	0.33	0.57	0.61	21
MI-3 (67-02D(7))	Line 11 67_02d7sp1prof1	0.17	1.11	0.33	0.71	0.67	0.50	0.34	0.57	0.61	23
MI-3 (67-02D(7))	Line 12 67_02d7sp1prof1	0.16	1.10	0.33	0.72	0.67	0.49	0.33	0.58	0.60	25
MI-3 (67-02D(7))	Line 13 67_02d7sp1prof1	0.16	1.13	0.33	0.70	0.67	0.50	0.33	0.57	0.62	28
MI-3 (67-02D(7))	Line 14 67_02d7sp1prof1	0.16	1.23	0.35	0.60	0.65	0.50	0.31	0.56	0.67	29
MI-3 (67-02D(7))	Line 15 67_02d7sp1prof1	0.15	1.29	0.35	0.55	0.65	0.50	0.30	0.56	0.70	31
MI-3 (67-02D(7))	Line 16 67_02d7sp1prof1	0.15	1.29	0.35	0.55	0.64	0.50	0.30	0.56	0.70	35
MI-3 (67-02D(7))	Line 17 67_02d7sp1prof1	0.14	1.30	0.36	0.55	0.64	0.50	0.29	0.56	0.70	37
MI-3 (67-02D(7))	Line 18 67_02d7sp1prof1	0.14	1.30	0.35	0.54	0.64	0.50	0.29	0.56	0.71	39
MI-3 (67-02D(7))	Line 19 67_02d7sp1prof1	0.15	1.30	0.35	0.54	0.64	0.50	0.30	0.56	0.71	41
MI-3 (67-02D(7))	Line 20 67_02d7sp1prof1	0.15	1.30	0.35	0.54	0.65	0.50	0.30	0.56	0.71	44
MI-3 (67-02D(7))	Line 21 67_02d7sp1prof1	0.14	1.30	0.35	0.55	0.64	0.50	0.29	0.56	0.70	46
MI-3 (67-02D(7))	Line 22 67_02d7sp1prof1	0.15	1.30	0.35	0.54	0.65	0.50	0.30	0.57	0.71	48
MI-3 (67-02D(7))	Line 23 67_02d7sp1prof1	0.15	1.30	0.35	0.54	0.64	0.50	0.30	0.56	0.71	51
MI-3 (67-02D(7))	Line 24 67_02d7sp1prof1	0.15	1.30	0.35	0.54	0.64	0.50	0.30	0.56	0.71	53
**Profile 4 (Raman spectrum for the corresponding inclusion is shown in Fig. 2i)**											
MI-4 (67-02D(7))	Line 3 67_02d7sp1prof2	0.18	1.10	0.31	0.70	0.68	0.49	0.37	0.58	0.61	6
MI-4 (67-02D(7))	Line 4 67_02d7sp1prof2	0.17	1.10	0.33	0.72	0.67	0.50	0.34	0.57	0.60	9
MI-4 (67-02D(7))	Line 5 67_02d7sp1prof2	0.17	1.05	0.32	0.77	0.67	0.50	0.35	0.58	0.58	12

MI-4 (67-02D(7))	Line 6 67_02d7sp1prof2	0.17	1.06	0.33	0.76	0.67	0.50	0.34	0.57	0.58	15
MI-4 (67-02D(7))	Line 7 67_02d7sp1prof2	0.17	1.07	0.33	0.75	0.67	0.49	0.34	0.58	0.59	18
MI-4 (67-02D(7))	Line 8 67_02d7sp1prof2	0.16	1.07	0.33	0.76	0.67	0.49	0.33	0.57	0.59	21
MI-4 (67-02D(7))	Line 9 67_02d7sp1prof2	0.16	1.07	0.33	0.76	0.67	0.49	0.33	0.58	0.58	24
MI-4 (67-02D(7))	Line 10 67_02d7sp1prof2	0.16	1.10	0.33	0.73	0.66	0.50	0.33	0.57	0.60	27
MI-4 (67-02D(7))	Line 11 67_02d7sp1prof2	0.16	1.24	0.35	0.59	0.64	0.51	0.31	0.56	0.68	30
MI-4 (67-02D(7))	Line 12 67_02d7sp1prof2	0.15	1.30	0.35	0.54	0.64	0.50	0.30	0.56	0.71	33
MI-4 (67-02D(7))	Line 13 67_02d7sp1prof2	0.15	1.31	0.36	0.54	0.64	0.51	0.29	0.56	0.71	36
MI-4 (67-02D(7))	Line 14 67_02d7sp1prof2	0.15	1.30	0.35	0.54	0.64	0.50	0.30	0.56	0.70	39
MI-4 (67-02D(7))	Line 15 67_02d7sp1prof2	0.15	1.29	0.35	0.54	0.64	0.50	0.30	0.56	0.70	42
MI-4 (67-02D(7))	Line 16 67_02d7sp1prof2	0.16	1.30	0.35	0.54	0.65	0.50	0.31	0.56	0.71	45
MI-4 (67-02D(7))	Line 17 67_02d7sp1prof2	0.15	1.29	0.36	0.55	0.64	0.51	0.30	0.56	0.70	48
MI-4 (67-02D(7))	Line 18 67_02d7sp1prof2	0.15	1.29	0.36	0.55	0.64	0.51	0.30	0.56	0.70	51
MI-4 (67-02D(7))	Line 19 67_02d7sp1prof2	0.15	1.29	0.35	0.55	0.64	0.50	0.30	0.56	0.70	54
MI-4 (67-02D(7))	Line 20 67_02d7sp1prof2	0.15	1.30	0.35	0.55	0.64	0.50	0.30	0.56	0.70	57
MI-4 (67-02D(7))	Line 21 67_02d7sp1prof2	0.15	1.30	0.34	0.54	0.65	0.49	0.31	0.57	0.71	60
MI-4 (67-02D(7))	Line 22 67_02d7sp1prof2	0.15	1.30	0.33	0.54	0.66	0.48	0.31	0.58	0.71	63
MI-4 (67-02D(7))	Line 24 67_02d7sp1prof2	0.16	1.30	0.33	0.54	0.67	0.48	0.32	0.58	0.71	69
MI-4 (67-02D(7))	Line 25 67_02d7sp1prof2	0.16	1.30	0.34	0.54	0.66	0.49	0.31	0.57	0.71	72
MI-4 (67-02D(7))	Line 26 67_02d7sp1prof2	0.15	1.30	0.34	0.54	0.65	0.49	0.30	0.57	0.71	75
MI-4 (67-02D(7))	Line 27 67_02d7sp1prof2	0.14	1.30	0.35	0.55	0.64	0.49	0.29	0.56	0.70	78
MI-4 (67-02D(7))	Line 28 67_02d7sp1prof2	0.15	1.30	0.35	0.55	0.65	0.50	0.30	0.56	0.70	81

Data from Bénard *et al.*[4]

Major element analyses by EPMA with spinel secondary standards analysed by Mössbauer (shown in Fig. 3d)

Thin horizontal lines separate excluded (high and variable $Fe^{3+}/\sum Fe$ next to melt inclusions) from selected (relatively low and constant $Fe^{3+}/\sum Fe$ far from melt inclusions) data to calculate fO_2 recorded in the West Bismarck sub-arc mantle peridotite in this study

[a] $\sum Fe = Fe^{2+} + Fe^{3+}$

[b] $Mg/(Mg+Fe_t)$

[c] Cr/(Cr+Al)

[d] Distance from melt inclusions (in microns)

Supplementary Table 7 | Major element abundances (wt%) in spinel standards measured during the same sessions as spinel in the Kamchatka and West Bismarck sub-arc mantle peridotites in this study

Sample	SiO_2	TiO_2	Al_2O_3	Cr_2O_3	FeO_t[a]	MnO	MgO	NiO	Total	$Fe^{3+}/\sum Fe$[b]
KLB8311	0.07	0.13	25.47	44.32	13.80	0.19	16.62	0.16	100.75	0.21
KLB8305	0.06	0.11	57.79	9.71	10.52	0.09	21.23	0.38	99.88	0.20
MBR8309	0.06	0.13	53.82	12.79	11.90	0.11	21.02	0.37	100.20	0.29
Mo4334_14	0.07	0.16	62.15	4.74	10.48	0.09	21.59	0.49	99.77	0.16
KLB8315	0.06	0.12	58.76	8.85	10.18	0.10	21.40	0.37	99.83	0.15
KLB8316	0.08	0.41	33.49	34.34	14.21	0.18	17.84	0.23	100.78	0.25

Major element analyses by EPMA

[a]All Fe treated as Fe^{2+}

[b]$Fe^{2+}/(Fe^{2+}+Fe^{3+})$ determined by Mössbauer spectroscopy by Wood & Virgo[30]

Supplementary Table 8 | Calculated stoichiometric compositions (At%) of spinel near melt pockets (MP) in the Kamchatka sub-arc mantle peridotite in this study

Sample	Analysis	Fe^{3+}	Cr	Fe^{2+}	Al	Mg	∑Fe[a]	Fe^{3+}/∑Fe	Mg#[b]	Cr#[c]	Distance[d] (μm)
**Profile 1**											
MP (Av33)	28 / 1 .	0.13	1.07	0.38	0.77	0.63	0.51	0.26	0.55	0.58	2
MP (Av33)	28 / 2 .	0.13	1.09	0.37	0.76	0.64	0.50	0.26	0.56	0.59	4
MP (Av33)	28 / 3 .	0.13	1.09	0.37	0.76	0.64	0.50	0.26	0.56	0.59	6
MP (Av33)	28 / 4 .	0.14	1.07	0.36	0.77	0.65	0.51	0.28	0.56	0.58	8
MP (Av33)	28 / 5 .	0.14	1.03	0.36	0.80	0.65	0.50	0.28	0.56	0.56	10
MP (Av33)	28 / 6 .	0.15	0.98	0.35	0.86	0.66	0.50	0.30	0.57	0.53	12
MP (Av33)	28 / 7 .	0.13	1.01	0.36	0.84	0.65	0.49	0.26	0.57	0.55	14
MP (Av33)	28 / 8 .	0.13	1.09	0.37	0.76	0.64	0.50	0.26	0.56	0.59	16
MP (Av33)	28 / 9 .	0.14	1.16	0.38	0.68	0.63	0.52	0.28	0.55	0.63	18
MP (Av33)	28 / 10 .	0.14	1.21	0.39	0.63	0.62	0.53	0.26	0.54	0.66	20
MP (Av33)	28 / 11 .	0.13	1.25	0.39	0.61	0.61	0.52	0.26	0.54	0.67	22
MP (Av33)	28 / 12 .	0.13	1.27	0.39	0.58	0.61	0.53	0.26	0.54	0.69	24
MP (Av33)	28 / 13 .	0.14	1.27	0.39	0.57	0.62	0.53	0.28	0.54	0.69	26
MP (Av33)	28 / 14 .	0.14	1.29	0.39	0.56	0.61	0.53	0.28	0.54	0.70	28
MP (Av33)	28 / 15 .	0.15	1.28	0.39	0.56	0.62	0.53	0.30	0.54	0.70	30
MP (Av33)	28 / 16 .	0.13	1.31	0.39	0.55	0.62	0.52	0.26	0.54	0.70	32
MP (Av33)	28 / 17 .	0.14	1.29	0.40	0.56	0.61	0.54	0.26	0.53	0.70	34
MP (Av33)	28 / 18 .	0.15	1.29	0.39	0.54	0.62	0.54	0.28	0.53	0.71	36
**Profile 2**											
MP (Av33)	29 / 2 .	0.14	1.09	0.38	0.73	0.64	0.52	0.27	0.55	0.60	2
MP (Av33)	29 / 3 .	0.14	1.10	0.37	0.74	0.64	0.51	0.28	0.56	0.60	4
MP (Av33)	29 / 4 .	0.15	1.08	0.36	0.75	0.65	0.51	0.29	0.56	0.59	6
MP (Av33)	29 / 5 .	0.15	1.07	0.36	0.76	0.65	0.51	0.29	0.56	0.58	8
MP (Av33)	29 / 6 .	0.15	1.06	0.36	0.77	0.66	0.50	0.30	0.57	0.58	10
MP (Av33)	29 / 7 .	0.16	1.08	0.36	0.75	0.65	0.52	0.30	0.56	0.59	12
MP (Av33)	29 / 8 .	0.15	1.12	0.36	0.71	0.65	0.51	0.30	0.56	0.61	14
MP (Av33)	29 / 9 .	0.14	1.16	0.38	0.68	0.63	0.52	0.27	0.55	0.63	16
MP (Av33)	29 / 10 .	0.14	1.21	0.37	0.64	0.63	0.51	0.27	0.55	0.66	18
MP (Av33)	29 / 11 .	0.14	1.25	0.38	0.60	0.63	0.52	0.27	0.55	0.68	20

MP (Av33)	29 / 12 .	0.14	1.26	0.39	0.58	0.62	0.53	0.27	0.54	0.69	22
MP (Av33)	29 / 13 .	0.13	1.30	0.39	0.56	0.61	0.52	0.24	0.54	0.70	24
MP (Av33)	29 / 14 .	0.15	1.28	0.38	0.56	0.62	0.53	0.27	0.54	0.70	26
MP (Av33)	29 / 15 .	0.13	1.29	0.40	0.56	0.61	0.53	0.25	0.54	0.70	28
MP (Av33)	29 / 16 .	0.14	1.30	0.39	0.56	0.62	0.53	0.26	0.54	0.70	30
MP (Av33)	29 / 17 .	0.13	1.29	0.39	0.57	0.62	0.52	0.26	0.54	0.69	32
MP (Av33)	29 / 18 .	0.14	1.28	0.40	0.57	0.61	0.53	0.26	0.53	0.69	34
**Profile 3**											
MP (Av33)	30 / 2 .	0.13	1.12	0.38	0.73	0.63	0.51	0.25	0.55	0.60	2
MP (Av33)	30 / 3 .	0.14	1.09	0.38	0.75	0.63	0.51	0.26	0.55	0.59	4
MP (Av33)	30 / 4 .	0.13	1.08	0.38	0.77	0.64	0.51	0.26	0.56	0.58	6
MP (Av33)	30 / 5 .	0.14	1.05	0.37	0.79	0.64	0.51	0.27	0.56	0.57	8
MP (Av33)	30 / 6 .	0.14	1.07	0.37	0.77	0.64	0.51	0.28	0.56	0.58	10
MP (Av33)	30 / 7 .	0.15	1.08	0.37	0.76	0.64	0.52	0.28	0.55	0.59	12
MP (Av33)	30 / 8 .	0.14	1.12	0.38	0.72	0.63	0.52	0.27	0.55	0.61	14
MP (Av33)	30 / 9 .	0.12	1.19	0.39	0.67	0.62	0.51	0.24	0.55	0.64	16
MP (Av33)	30 / 10 .	0.15	1.19	0.38	0.65	0.63	0.53	0.28	0.54	0.65	18
MP (Av33)	30 / 11 .	0.13	1.22	0.39	0.64	0.62	0.52	0.25	0.54	0.66	20
MP (Av33)	30 / 12 .	0.13	1.21	0.39	0.64	0.62	0.52	0.26	0.54	0.66	22
MP (Av33)	30 / 13 .	0.14	1.22	0.39	0.62	0.62	0.53	0.27	0.54	0.66	24
MP (Av33)	30 / 14 .	0.14	1.23	0.39	0.62	0.62	0.53	0.26	0.54	0.66	26
MP (Av33)	30 / 15 .	0.12	1.24	0.39	0.62	0.61	0.52	0.24	0.54	0.67	28
MP (Av33)	30 / 16 .	0.13	1.23	0.39	0.62	0.62	0.52	0.25	0.54	0.66	30
MP (Av33)	30 / 17 .	0.12	1.25	0.39	0.62	0.61	0.51	0.23	0.54	0.67	32
MP (Av33)	30 / 18 .	0.14	1.23	0.38	0.61	0.63	0.52	0.27	0.55	0.67	34
MP (Av33)	30 / 19 .	0.13	1.25	0.40	0.60	0.60	0.53	0.25	0.53	0.68	36
MP (Av33)	30 / 20 .	0.13	1.24	0.40	0.61	0.61	0.53	0.25	0.53	0.67	38
**Profile 4**											
MP (Av33)	174 / 1 .	0.15	1.06	0.36	0.76	0.64	0.51	0.29	0.56	0.58	2
MP (Av33)	174 / 2 .	0.14	1.06	0.37	0.78	0.63	0.51	0.27	0.55	0.58	4
MP (Av33)	174 / 3 .	0.15	1.05	0.36	0.79	0.64	0.51	0.29	0.56	0.57	6
MP (Av33)	174 / 4 .	0.14	1.04	0.36	0.80	0.64	0.50	0.28	0.56	0.57	8
MP (Av33)	174 / 5 .	0.15	1.10	0.37	0.73	0.63	0.52	0.29	0.55	0.60	10

MP (Av33)	174 / 6 .	0.16	1.19	0.37	0.64	0.63	0.53	0.30	0.54	0.65	12
MP (Av33)	174 / 7 .	0.14	1.21	0.39	0.64	0.62	0.53	0.26	0.54	0.65	14
MP (Av33)	174 / 8 .	0.14	1.21	0.39	0.64	0.61	0.53	0.26	0.54	0.65	16
MP (Av33)	174 / 9 .	0.13	1.24	0.39	0.61	0.60	0.53	0.25	0.53	0.67	18
MP (Av33)	174 / 10 .	0.14	1.25	0.38	0.60	0.62	0.52	0.27	0.54	0.68	20
MP (Av33)	174 / 11 .	0.15	1.27	0.39	0.57	0.61	0.54	0.28	0.53	0.69	22
MP (Av33)	174 / 12 .	0.14	1.29	0.40	0.56	0.60	0.54	0.26	0.52	0.70	24
MP (Av33)	174 / 13 .	0.15	1.28	0.40	0.56	0.60	0.54	0.27	0.52	0.70	26
MP (Av33)	174 / 14 .	0.15	1.28	0.39	0.56	0.60	0.54	0.28	0.53	0.69	28
MP (Av33)	174 / 15 .	0.14	1.28	0.39	0.58	0.60	0.53	0.26	0.53	0.69	30

Data from Ionov *et al.*[3]

Major element analyses by EPMA (shown in Supplementary Fig. 8)

[a]$\sum Fe = Fe^{2+} + Fe^{3+}$

[b]$Mg/(Mg+Fe_t)$

[c]$Cr/(Cr+Al)$

[d]Distance from melt pockets (in microns)

Supplementary Table 9 | Average major element abundances (wt%) in olivine and orthopyroxene in the Kamchatka and West Bismarck sub-arc mantle peridotites in this study used to calculate their temperatures and fO_2

Sample	SiO_2	TiO_2	Al_2O_3	Cr_2O_3	FeO_t[a]	MnO	MgO	CaO	Na_2O	K_2O	NiO	P_2O_5	Total
**Avacha (Av33)**													
Olivine	40.48	0.02	0.01	0.01	9.14	0.15	49.40	0.06	0.01	0.00	0.39	0.01	99.69
Error (1σ)	0.22	0.02	0.01	0.01	0.15	0.01	0.23	0.01	0.01	0.01	0.02	0.01	0.30
Opx	56.45	0.04	1.36	0.44	6.12	0.17	34.31	0.81	0.02	0.01	0.09	0.01	99.81
Error (1σ)	0.37	0.03	0.40	0.09	0.13	0.03	0.55	0.28	0.01	0.01	0.03	0.01	0.34
**Ritter (67-02D(7))**													
Olivine	41.25	–	–	0.00	7.94	0.12	51.00	0.02	–	–	0.41	–	100.75
Error (1σ)	0.17	–	–	0.00	0.17	0.01	0.12	0.00	–	–	0.02	–	0.19
Opx	57.34	0.01	1.34	0.47	5.45	0.14	35.13	0.52	0.01	–	–	–	100.41
Error (1σ)	0.25	0.01	0.11	0.02	0.03	0.00	0.16	0.15	0.01	–	–	–	0.35

Data from Bénard *et al.*[2]

Opx, orthopyroxene

[a]All Fe treated as Fe^{2+}

Supplementary Table 10 | Average major element abundances (At%) in spinel in the Kamchatka and West Bismarck sub-arc mantle peridotites in this study used to calculate their temperatures and fO_2

Sample	$Fe^{3+}_{corrected}$	Cr	$Fe^{2+}_{corrected}$	Al	Mg	$\sum Fe_{corrected}$[a]	$Fe^{3+}/\sum Fe_{corrected}$	Mg#[b]	Cr#[c]	T (°C)[d]	$\Delta \log fO_2$[FMQ][e]	$\Delta \log fO_2$[FMQ][f]
**Avacha (Av33)**												
Average	0.15	1.23	0.36	0.60	0.64	0.51	0.30	0.56	0.67	1016	1.04	0.72
Error (1σ)	0.01	0.03	0.01	0.03	0.01	0.01	0.01	0.01	0.02	25	0.23	0.19
**Ritter (67-02D(7))**[g]												
Average	0.15	1.29	0.36	0.56	0.64	0.50	0.29	0.56	0.70	955	1.39	0.90
Error (1σ)	0.01	0.02	0.01	0.02	0.01	0.01	0.01	0.01	0.01	28	0.28	0.24

The average major element compositions of spinel are calculated using the portions away from inclusions of profiles (stable composition) given in Supplementary Tables 5 & 6

[a]$\sum Fe = Fe^{2+} + Fe^{3+}$

[b]$Mg/(Mg+Fe_t)$

[c]Cr/(Cr+Al)

[d]Temperatures calculated with the olivine-spinel geothermometer of Ballhaus *et al.*[28] at 1.5 GPa

[e]fO_2 calculated using the olivine-spinel temperature of Ballhaus *et al.*[28] and the oxybarometer of Wood *et al.*[27] at 1.5 Gpa (shown in Fig. 1a)

[f]fO_2 calculated using the olivine-spinel temperature and the oxybarometer of Ballhaus *et al.*[28] at 1.5 GPa

[g]Data from Bénard *et al.*[4]

Supplementary Table 11 | Major element (wt%) and sulfur (ppm) abundances in and isotope compositions ($\delta^{34}S_{measured}$, ‰) of standard JDF 46°N and melt pockets (MP) in the Kamchatka sub-arc mantle peridotite in this study

Sample	SiO_2	TiO_2	Al_2O_3	Cr_2O_3	FeO_t[a]	MnO	MgO	CaO	Na_2O	K_2O	P_2O_5	S	Mg#[b]	Fe/S[c]	$S^{6+}/\sum S$[d]	Total	$(^{34}S/^{32}S)_{corrected}$	IMF[e]	$\delta^{34}S_{measured}$[f]	Error (2σ)
JDF 46°N (standard)[g]	50.01	1.97	13.70	–	12.25	0.21	6.90	10.81	2.64	0.17	0.19	1121	–	–	–	99.13	–	–	–	–
Error (1σ)[g]	0.46	0.04	0.07	–	0.10	0.02	0.09	0.10	0.05	0.01	0.02	65	–	–	–	–	–	–	–	–
**Analyses**																				
JDF 46°N (standard)	–	–	–	–	–	–	–	–	–	–	–	–	–	–	–	–	0.04398	0.9945	1.3	0.60
JDF 46°N (standard)	–	–	–	–	–	–	–	–	–	–	–	–	–	–	–	–	0.04401	0.9951	1.3	0.62
JDF 46°N (standard)	–	–	–	–	–	–	–	–	–	–	–	–	–	–	–	–	0.04399	0.9949	1.3	0.57
MP-1 (unknown, Av33)	59.72	0.47	18.53	0.28	3.25	b.d.	3.37	7.15	4.50	0.65	0.26	1908	0.65	7.61	-	98.57	0.04429	0.9944	8.5	0.85
MP-2 (unknown, Av33)	60.16	0.72	18.80	0.31	3.09	0.08	3.85	7.99	4.31	0.76	0.21	1713	0.69	8.05	-	100.63	0.04436	0.9944	10.1	0.90
MP-3 (unknown, Av33)	60.28	0.71	18.40	0.89	3.13	0.09	3.48	7.70	4.49	0.54	0.32	1333	0.66	10.5	-	100.28	0.04432	0.9944	9.1	0.60
JDF 46°N (standard)	–	–	–	–	–	–	–	–	–	–	–	–	–	–	–	–	0.04394	0.9936	1.3	0.53
JDF 46°N (standard)	–	–	–	–	–	–	–	–	–	–	–	–	–	–	–	–	0.04397	0.9943	1.3	0.54
MP-4 (unknown, Av33)	*60.73*	*0.40*	*19.24*	*0.39*	*3.45*	*0.03*	*3.34*	*7.97*	*4.23*	*0.68*	*0.26*	*1147*	*0.63*	*13.4*	*0.55–0.58*	*100.95*	*0.04436*	*0.9942*	*10*	*1.04*
MP-5 (unknown, Av33)	60.44	0.37	18.71	0.88	3.25	0.01	3.36	7.08	4.28	0.73	0.11	1145	0.65	12.7	–	99.44	0.04439	0.9942	11.0	0.98
JDF 46°N (standard)	–	–	–	–	–	–	–	–	–	–	–	–	–	–	–	–	0.04397	0.9943	1.3	0.60
MP-6 (unknown, Av33)	60.40	0.17	18.22	0.37	3.04	0.07	3.03	7.06	4.67	0.74	0.42	1338	0.64	10.1	–	98.46	0.04426	0.9937	8.5	0.99
MP-7 (unknown, Av33)	60.65	0.42	18.82	0.44	2.79	0.01	2.82	6.81	4.61	0.70	0.11	764	0.64	16.3	–	98.32	0.04419	0.9937	7	1.60
JDF 46°N (standard)	–	–	–	–	–	–	–	–	–	–	–	–	–	–	–	–	0.04388	0.9923	1.4	0.57
JDF 46°N (standard)	–	–	–	–	–	–	–	–	–	–	–	–	–	–	–	–	0.04394	0.9937	1.3	0.60

Major element analyses by EPMA and S isotopes analysed by SIMS (shown in Fig. 4b)

Analysis in italics is that of the MP also characterised by Raman spectrometry (shown in Fig. 2i)

[a]All Fe treated as Fe^{2+}

[b]$Mg/(Mg+Fe_t)$

[c]Molar ratio

[d]$\sum S=S^{2-}+S^{6+}$ (XANES data, shown in Fig. 2c, f)

[e]Instrumental mass fractionation

[f]$((^{34}S/^{32}S)_{corrected}/IMF/0.04416375-1)\times1000$

[g]Data from Fiege *et al.*[31]; this standard has a $\delta^{34}S_{true}$ of 1.3

Supplementary Table 12 | Calculated major element abundances (wt%) for low-temperature (LT) inclusions and melt pockets (MP) at variable fO_2 and $Fe^{3+}/\sum Fe$ in spinel at equilibrium

$\Delta \log fO_2$[FMQ]	SiO_2	TiO_2	Al_2O_3	Fe_2O_3	FeO	MnO	MgO	NiO	CaO	Na_2O	K_2O	P_2O_5	H_2O	$Fe^{3+}/\sum Fe$[a]	$Fe^{3+}/\sum Fe$[b]
**LT inclusions (0.2 GPa, 1050°C)**[c]															
-3	59.58	0.28	19.78	0.07	1.33	0.06	0.52	0.04	7.70	3.27	0.47	0.16	6.74	0.04	0.10
-2.5	59.58	0.28	19.78	0.09	1.32	0.06	0.52	0.04	7.70	3.27	0.47	0.16	6.74	0.05	0.13
-2	59.58	0.28	19.78	0.11	1.30	0.06	0.52	0.04	7.70	3.27	0.47	0.16	6.74	0.07	0.16
-1.5	59.58	0.28	19.78	0.13	1.28	0.06	0.52	0.04	7.70	3.27	0.47	0.16	6.74	0.08	0.19
-1	59.58	0.28	19.78	0.16	1.25	0.06	0.52	0.04	7.70	3.27	0.47	0.16	6.74	0.10	0.22
-0.5	59.58	0.28	19.78	0.19	1.22	0.06	0.52	0.04	7.70	3.27	0.47	0.16	6.74	0.13	0.27
0	59.58	0.28	19.78	0.24	1.18	0.06	0.52	0.04	7.70	3.27	0.47	0.16	6.74	0.15	0.31
0.5	59.57	0.28	19.78	0.29	1.14	0.06	0.52	0.04	7.70	3.27	0.47	0.16	6.74	0.18	0.36
1	59.57	0.28	19.77	0.34	1.09	0.06	0.52	0.04	7.70	3.27	0.47	0.16	6.74	0.22	0.42
1.5	59.57	0.28	19.77	0.41	1.03	0.06	0.52	0.04	7.70	3.27	0.47	0.16	6.73	0.26	0.47
2	59.57	0.28	19.77	0.48	0.97	0.06	0.52	0.04	7.70	3.27	0.47	0.16	6.73	0.31	0.53
2.5	59.56	0.28	19.77	0.55	0.90	0.06	0.52	0.04	7.70	3.27	0.47	0.16	6.73	0.36	0.58
3	59.56	0.28	19.77	0.64	0.82	0.06	0.52	0.04	7.70	3.27	0.47	0.16	6.73	0.41	0.64
3.5	59.55	0.28	19.77	0.72	0.75	0.06	0.52	0.04	7.70	3.27	0.47	0.16	6.73	0.47	0.69
4	59.55	0.28	19.77	0.81	0.67	0.06	0.52	0.04	7.70	3.27	0.46	0.15	6.72	0.52	0.73
4.5	59.55	0.28	19.77	0.90	0.59	0.06	0.52	0.04	7.70	3.27	0.46	0.15	6.72	0.58	0.78
5	59.54	0.28	19.76	0.98	0.51	0.06	0.52	0.04	7.70	3.26	0.46	0.15	6.72	0.63	0.81
**LT inclusions (1 GPa, 1050°C)**															
-3	58.20	0.27	19.32	0.05	1.32	0.06	0.50	0.04	7.52	3.19	0.45	0.15	8.92	0.03	0.07
-2.5	58.20	0.27	19.32	0.06	1.31	0.06	0.50	0.04	7.52	3.19	0.45	0.15	8.92	0.04	0.09
-2	58.20	0.27	19.32	0.07	1.30	0.06	0.50	0.04	7.52	3.19	0.45	0.15	8.92	0.05	0.11
-1.5	58.20	0.27	19.32	0.09	1.28	0.06	0.50	0.04	7.52	3.19	0.45	0.15	8.92	0.06	0.14
-1	58.19	0.27	19.32	0.11	1.26	0.06	0.50	0.04	7.52	3.19	0.45	0.15	8.92	0.07	0.17
-0.5	58.19	0.27	19.32	0.14	1.24	0.06	0.50	0.04	7.52	3.19	0.45	0.15	8.92	0.09	0.20
0	58.19	0.27	19.32	0.17	1.21	0.06	0.50	0.04	7.52	3.19	0.45	0.15	8.92	0.11	0.24
0.5	58.19	0.27	19.32	0.20	1.18	0.06	0.50	0.04	7.52	3.19	0.45	0.15	8.92	0.13	0.28
1	58.19	0.27	19.31	0.25	1.14	0.06	0.50	0.04	7.52	3.19	0.45	0.15	8.92	0.16	0.33
1.5	58.18	0.27	19.31	0.30	1.10	0.06	0.50	0.04	7.52	3.19	0.45	0.15	8.91	0.20	0.38
2	58.18	0.27	19.31	0.35	1.04	0.06	0.50	0.04	7.52	3.19	0.45	0.15	8.91	0.23	0.43
2.5	58.18	0.27	19.31	0.42	0.99	0.06	0.50	0.04	7.52	3.19	0.45	0.15	8.91	0.28	0.49
3	58.17	0.27	19.31	0.49	0.92	0.06	0.50	0.04	7.52	3.19	0.45	0.15	8.91	0.32	0.55
3.5	58.17	0.27	19.31	0.57	0.85	0.06	0.50	0.04	7.52	3.19	0.45	0.15	8.91	0.37	0.60
4	58.16	0.27	19.31	0.65	0.78	0.06	0.50	0.04	7.52	3.19	0.45	0.15	8.91	0.43	0.65
4.5	58.16	0.27	19.31	0.73	0.70	0.06	0.50	0.04	7.52	3.19	0.45	0.15	8.91	0.48	0.70
5	58.15	0.27	19.30	0.82	0.63	0.06	0.50	0.04	7.52	3.19	0.45	0.15	8.91	0.54	0.75

**LT inclusions (1.5 GPa, 1050°C)**															
-3	58.20	0.27	19.32	0.04	1.33	0.06	0.50	0.04	7.52	3.19	0.45	0.15	8.92	0.03	0.06
-2.5	58.20	0.27	19.32	0.05	1.32	0.06	0.50	0.04	7.52	3.19	0.45	0.15	8.92	0.03	0.08
-2	58.20	0.27	19.32	0.06	1.31	0.06	0.50	0.04	7.52	3.19	0.45	0.15	8.92	0.04	0.09
-1.5	58.20	0.27	19.32	0.07	1.30	0.06	0.50	0.04	7.52	3.19	0.45	0.15	8.92	0.05	0.11
-1	58.20	0.27	19.32	0.09	1.28	0.06	0.50	0.04	7.52	3.19	0.45	0.15	8.92	0.06	0.14
-0.5	58.19	0.27	19.32	0.11	1.26	0.06	0.50	0.04	7.52	3.19	0.45	0.15	8.92	0.07	0.17
0	58.19	0.27	19.32	0.14	1.24	0.06	0.50	0.04	7.52	3.19	0.45	0.15	8.92	0.09	0.20
0.5	58.19	0.27	19.32	0.17	1.21	0.06	0.50	0.04	7.52	3.19	0.45	0.15	8.92	0.11	0.24
1	58.19	0.27	19.32	0.21	1.18	0.06	0.50	0.04	7.52	3.19	0.45	0.15	8.92	0.14	0.28
1.5	58.19	0.27	19.31	0.25	1.14	0.06	0.50	0.04	7.52	3.19	0.45	0.15	8.92	0.16	0.33
2	58.18	0.27	19.31	0.30	1.09	0.06	0.50	0.04	7.52	3.19	0.45	0.15	8.91	0.20	0.38
2.5	58.18	0.27	19.31	0.36	1.04	0.06	0.50	0.04	7.52	3.19	0.45	0.15	8.91	0.24	0.44
3	58.18	0.27	19.31	0.42	0.98	0.06	0.50	0.04	7.52	3.19	0.45	0.15	8.91	0.28	0.49
3.5	58.17	0.27	19.31	0.50	0.92	0.06	0.50	0.04	7.52	3.19	0.45	0.15	8.91	0.33	0.55
4	58.17	0.27	19.31	0.57	0.85	0.06	0.50	0.04	7.52	3.19	0.45	0.15	8.91	0.38	0.61
4.5	58.16	0.27	19.31	0.66	0.77	0.06	0.50	0.04	7.52	3.19	0.45	0.15	8.91	0.43	0.66
5	58.16	0.27	19.31	0.74	0.70	0.06	0.50	0.04	7.52	3.19	0.45	0.15	8.91	0.49	0.71
**MP (0.2 GPa, 1250°C)**															
-3	60.60	0.44	18.78	0.15	2.89	0.07	3.26	0.04	7.75	4.46	0.74	0.23	0.59	0.04	0.10
-2.5	60.59	0.44	18.78	0.18	2.86	0.07	3.26	0.04	7.75	4.46	0.74	0.23	0.59	0.05	0.13
-2	60.59	0.44	18.77	0.22	2.82	0.07	3.26	0.04	7.75	4.46	0.74	0.23	0.59	0.07	0.15
-1.5	60.59	0.44	18.77	0.27	2.77	0.07	3.26	0.04	7.75	4.46	0.74	0.23	0.59	0.08	0.18
-1	60.58	0.44	18.77	0.34	2.72	0.07	3.26	0.04	7.75	4.46	0.74	0.23	0.59	0.10	0.22
-0.5	60.58	0.44	18.77	0.41	2.65	0.07	3.26	0.04	7.75	4.46	0.74	0.23	0.59	0.12	0.26
0	60.57	0.44	18.77	0.50	2.57	0.07	3.26	0.04	7.75	4.46	0.74	0.23	0.59	0.15	0.31
0.5	60.57	0.44	18.77	0.60	2.48	0.07	3.26	0.04	7.75	4.46	0.74	0.23	0.59	0.18	0.36
1	60.56	0.44	18.77	0.72	2.37	0.07	3.26	0.04	7.75	4.46	0.74	0.23	0.59	0.22	0.41
1.5	60.55	0.44	18.76	0.86	2.25	0.07	3.26	0.04	7.75	4.45	0.74	0.23	0.59	0.26	0.47
2	60.54	0.44	18.76	1.01	2.11	0.07	3.26	0.04	7.74	4.45	0.74	0.23	0.59	0.30	0.52
2.5	60.53	0.44	18.76	1.18	1.96	0.07	3.26	0.04	7.74	4.45	0.74	0.23	0.59	0.35	0.58
3	60.52	0.44	18.75	1.36	1.80	0.07	3.26	0.04	7.74	4.45	0.74	0.23	0.59	0.40	0.63
3.5	60.51	0.44	18.75	1.54	1.63	0.07	3.26	0.04	7.74	4.45	0.74	0.23	0.59	0.46	0.68
4	60.50	0.44	18.75	1.73	1.46	0.07	3.26	0.04	7.74	4.45	0.74	0.23	0.59	0.52	0.73
4.5	60.49	0.44	18.74	1.92	1.29	0.07	3.26	0.04	7.74	4.45	0.74	0.23	0.59	0.57	0.77
5	60.48	0.44	18.74	2.10	1.13	0.07	3.26	0.04	7.74	4.45	0.74	0.23	0.59	0.63	0.81
**MP (1 GPa, 1250°C)**															
-3	60.60	0.44	18.78	0.11	2.93	0.07	3.26	0.04	7.75	4.46	0.74	0.23	0.59	0.03	0.08
-2.5	60.60	0.44	18.78	0.13	2.90	0.07	3.26	0.04	7.75	4.46	0.74	0.23	0.59	0.04	0.09
-2	60.59	0.44	18.78	0.16	2.88	0.07	3.26	0.04	7.75	4.46	0.74	0.23	0.59	0.05	0.11

-1.5	60.59	0.44	18.78	0.20	2.84	0.07	3.26	0.04	7.75	4.46	0.74	0.23	0.59	0.06	0.14
-1	60.59	0.44	18.77	0.25	2.80	0.07	3.26	0.04	7.75	4.46	0.74	0.23	0.59	0.07	0.17
-0.5	60.59	0.44	18.77	0.31	2.75	0.07	3.26	0.04	7.75	4.46	0.74	0.23	0.59	0.09	0.20
0	60.58	0.44	18.77	0.37	2.68	0.07	3.26	0.04	7.75	4.46	0.74	0.23	0.59	0.11	0.24
0.5	60.58	0.44	18.77	0.46	2.61	0.07	3.26	0.04	7.75	4.46	0.74	0.23	0.59	0.14	0.28
1	60.57	0.44	18.77	0.55	2.52	0.07	3.26	0.04	7.75	4.46	0.74	0.23	0.59	0.16	0.33
1.5	60.56	0.44	18.77	0.67	2.42	0.07	3.26	0.04	7.75	4.46	0.74	0.23	0.59	0.20	0.38
2	60.56	0.44	18.76	0.79	2.31	0.07	3.26	0.04	7.75	4.46	0.74	0.23	0.59	0.24	0.44
2.5	60.55	0.44	18.76	0.94	2.18	0.07	3.26	0.04	7.75	4.45	0.74	0.23	0.59	0.28	0.49
3	60.54	0.44	18.76	1.10	2.03	0.07	3.26	0.04	7.74	4.45	0.74	0.23	0.59	0.33	0.55
3.5	60.53	0.44	18.76	1.27	1.88	0.07	3.26	0.04	7.74	4.45	0.74	0.23	0.59	0.38	0.61
4	60.52	0.44	18.75	1.45	1.71	0.07	3.26	0.04	7.74	4.45	0.74	0.23	0.59	0.43	0.66
4.5	60.50	0.44	18.75	1.64	1.54	0.07	3.26	0.04	7.74	4.45	0.74	0.23	0.59	0.49	0.71
5	60.49	0.44	18.74	1.83	1.37	0.07	3.26	0.04	7.74	4.45	0.74	0.23	0.59	0.55	0.75
**MP (1.5 GPa, 1250°C)**															
-3	60.60	0.44	18.78	0.09	2.94	0.07	3.26	0.04	7.75	4.46	0.74	0.23	0.59	0.03	0.06
-2.5	60.60	0.44	18.78	0.11	2.92	0.07	3.26	0.04	7.75	4.46	0.74	0.23	0.59	0.03	0.08
-2	60.60	0.44	18.78	0.14	2.90	0.07	3.26	0.04	7.75	4.46	0.74	0.23	0.59	0.04	0.10
-1.5	60.59	0.44	18.78	0.17	2.87	0.07	3.26	0.04	7.75	4.46	0.74	0.23	0.59	0.05	0.12
-1	60.59	0.44	18.78	0.21	2.83	0.07	3.26	0.04	7.75	4.46	0.74	0.23	0.59	0.06	0.14
-0.5	60.59	0.44	18.77	0.26	2.79	0.07	3.26	0.04	7.75	4.46	0.74	0.23	0.59	0.08	0.17
0	60.59	0.44	18.77	0.32	2.74	0.07	3.26	0.04	7.75	4.46	0.74	0.23	0.59	0.09	0.21
0.5	60.58	0.44	18.77	0.39	2.67	0.07	3.26	0.04	7.75	4.46	0.74	0.23	0.59	0.12	0.25
1	60.58	0.44	18.77	0.47	2.60	0.07	3.26	0.04	7.75	4.46	0.74	0.23	0.59	0.14	0.29
1.5	60.57	0.44	18.77	0.57	2.51	0.07	3.26	0.04	7.75	4.46	0.74	0.23	0.59	0.17	0.34
2	60.56	0.44	18.77	0.68	2.40	0.07	3.26	0.04	7.75	4.46	0.74	0.23	0.59	0.20	0.39
2.5	60.56	0.44	18.76	0.82	2.29	0.07	3.26	0.04	7.75	4.46	0.74	0.23	0.59	0.24	0.45
3	60.55	0.44	18.76	0.96	2.15	0.07	3.26	0.04	7.75	4.45	0.74	0.23	0.59	0.29	0.50
3.5	60.54	0.44	18.76	1.12	2.01	0.07	3.26	0.04	7.74	4.45	0.74	0.23	0.59	0.34	0.56
4	60.53	0.44	18.75	1.30	1.85	0.07	3.26	0.04	7.74	4.45	0.74	0.23	0.59	0.39	0.61
4.5	60.51	0.44	18.75	1.48	1.68	0.07	3.26	0.04	7.74	4.45	0.74	0.23	0.59	0.44	0.67
5	60.50	0.44	18.75	1.67	1.52	0.07	3.26	0.04	7.74	4.45	0.74	0.23	0.59	0.50	0.71

The compositions used for the calculations are averages of those of glasses in LT inclusions (homogenised at 900°C) and MP reported by Ionov *et al.*[3]. The H_2O contents are inferred from deficiency of totals of average glass analyses.

[a]$Fe^{3+}/(Fe^{2+}+Fe^{3+})$ in melt calculated with the model of Kress & Carmichael[24] using pMELTS[25]

[b]$Fe^{3+}/(Fe^{2+}+Fe^{3+})$ in spinel at equilibrium calculated with the partition coefficients of Mallmann & O'Neill[26] (shown in Fig. 1a)

[c]These melts are saturated in a pure H_2O phase at 0.2 GPa

Supplementary Table 13 | Average trace element abundances (wt% and ppm) in low-temperature (LT) inclusions, melt pockets (MP) and vein melt inclusions (MI) in the Kamchatka sub-arc mantle peridotites in this study

Element	LT[a]	Error (1σ)	MP[a]	Error (1σ)	Vein MI[b]	Error (1σ)
Al_2O_3 (wt%)	–	–	–	–	17.9	1.6
CaO (wt%)	8.0	0.3	8.0	0.1	4.8	2.2
Cs (ppm)	0.57	0.18	0.59	0.07	0.3	0.1
Rb	5	2	7.7	0.8	11	2
Ba	135	32	184	11	57	16
Th	1.2	0.8	0.8	0.2	b.d.	b.d.
U	1.1	0.4	b.d.	b.d.	b.d.	b.d.
Nb	1.1	0.7	1.0	0.4	1.5	0.6
La	3.9	0.8	4.0	0.8	0.9	0.4
Ce	8	1	10	2	1.6	0.5
Pb	14	9	4	2	b.d.	b.d.
Pr	1.3	0.2	1.5	0.3	0.16	0.07
Sr	325	48	394	15	79	19
Nd	7	1	8	2	0.5	0.3
Sm	3	1	2.6	0.9	0.08	0.04
Zr	41	7	52	3	11	4
Hf	2	2	1.9	0.1	0.25	0.09
Eu	1.0	0.4	0.9	0.5	0.03	0.01
Gd	3	1	2.3	0.4	0.08	0.02
Dy	2.3	0.8	2.4	0.4	0.17	0.08
Ho	0.5	0.1	0.5	0.1	0.05	0.02
Y	9	3	11.0	0.7	1.4	0.9
Er	1.4	0.5	1.3	0.2	0.2	0.1
Yb	1.7	0.6	1.4	0.5	0.3	0.2

b.d., below detection

[a]Data from Ionov *et al.*[3] (shown in Fig. 4b, U and Pb were not reported in the original publication)

[b]Data for dacite vein MI in Bénard *et al.*[5] (shown in Fig. 4b)

Supplementary Discussion

The samples investigated in this study are all sub-arc mantle-derived spinel harzburgite brought to the surface by recent volcanic activity at Avacha and Ritter volcanoes, located in Kamchatka (Russia) and the West Bismarck (Papua New Guinea) arcs respectively. The xenoliths are highly similar in the two localities and are all residual rocks formed by large degrees (28–35%) of fluxed melting in the mantle wedge, mostly at 1–2 GPa (refs 1,2). After the last melting event, the Kamchatka and West Bismarck melting residues were accreted to the sub-arc lithosphere where they experienced slow, long-term cooling to 900–1000°C and 650–800°C, respectively[1,2]. These equilibration conditions are positively correlated with the thickness of the overlying arc crust, which ranges from ~18 km in the West Bismarck Arc to ~35 km in the Kamchatka Arc[1,2]. Kamchatka and West Bismarck harzburgites contain glass (formerly melt) inclusions in spinel (chromite), either disseminated in the harzburgite matrices[2–4] or sometimes distributed in cross-cutting pyroxenite veins[5], which record the percolation of primitive subduction zone silicate melts through the sub-arc mantle lithosphere.

In this study, we examine two groups of disseminated inclusions in spinel from a Kamchatka sub-arc harzburgite xenolith previously studied by Ionov *et al.*[3] (Av33): LT inclusions and melt pockets (MP). The petrological and geochemical features of these two types of inclusions were originally investigated in detail, using three different spinel harzburgite samples (AP7, Av17 and Av33)[3]. On the contrary to vein MI (see below), no clear relationship between neither LT inclusions nor MP and a melt channel source (*e.g.* a vein) was identified at the sample scale; the selection of the three Kamchatka mantle xenoliths was based on the high abundances of glass inclusions in these samples[3].

Two main modes of occurrence of LT inclusions were observed: LT inclusions in 'trails' or rims in partially reacted spinel (see Fig. 2 in Ionov *et al.*[3] and Supplementary Fig. 7), and LT

inclusions entirely peppering strongly reacted spinel (Figs 2a & 3a). The LT inclusions contain only glass or glass and amphibole, sometimes with accessory sulfides[3] (Supplementary Figs 2–4, 6 & 7). The MP have irregular shapes, indicating that these formed in spinel at a later stage than LT inclusions, and generally do not contain daughter silicate minerals (see Figs 2, 3 & 5 in Ionov *et al.*[3]). The composition of spinel hosting LT inclusions and MP is always highly heterogeneous at the grain scale, which suggests that this phase formed during the re-crystallisation of pre-existing (*i.e.* of residual origin) grains in the presence of a pervasively percolating melt (see Figs 2 & 3 in Ionov *et al.*[3] and Fig. 3a, b & Supplementary Fig. 7).

The net rounded shape displayed by all the MI in Ionov *et al.*[3] supports their formation at high pressure and temperature in the mantle, for instance in contrast with the 'sieve' mineral-melt texture developed during recent interactions between peridotite xenoliths and their carrier magmas at sub-surface conditions[4,6]. The texture of LT inclusions and MP is also not consistent with an origin related to the late-stage percolation of the carrier magma in the deep crust, as this has been rarely but clearly identified in some Kamchatka mantle xenoliths (from the same collection as those from Ionov *et al.*[3]) with the formation of Fe-rich amphibole veins intruding brittle fractures[7]. Based on a comparison of the compositions of amphibole in the carrier magma, the Fe-rich veins and in LT inclusions, it has been demonstrated that these are not related to each other[3].

After heating experiments were conducted by Ionov *et al.*[3] to homogenise LT inclusions (*i.e.* dissolve the possibly occurring daughter amphibole), it was further established by these authors that the distinct melt compositional fields formed by LT inclusions and MP could not be related to the pervasive infiltration of the host andesite magma (see Fig. 6 in Ionov *et al.*[3] and Supplementary Fig. 1). Finally, the trace element abundances in both glasses of LT inclusions and MP are too depleted to be formed by percolation of the carrier magma in the xenoliths, and suggest instead a more primitive origin (see Fig. 8 in Ionov *et al.*[3] and Fig. 4a).

All XANES, Raman spectrometry and EPMA data reported in this study for the determination of S and Fe valence states, respectively in LT inclusions and MP and their host spinel, have been acquired on spinel grains from sample Av33 that have not been experimentally treated (*i.e.* unheated). Only Kamchatka inclusions found 'naturally homogeneous', *i.e.* free of daughter silicate minerals such as amphibole in LT inclusions, were investigated here. Subsequently to the study by Ionov *et al.*[3], Bénard *et al.*[2] have reported the major element compositions of olivine and orthopyroxene from sample Av33, for which equilibrium oxygen fugacity (fO_2) is calculated here (Fig. 1a). These compositions are given in Supplementary Table 9.

In this study, we also examine spinel-hosted MI in amphibole-bearing websterite veins (vein MI) crosscutting two (Av24 and Av25) of the three spinel harzburgite xenoliths from Avacha previously studied by Bénard *et al.*[5] (Av23, Av24 and Av25). These veins are similar to those reported earlier by Halama *et al.*[8]. All the vein MI are hosted by abundant spinel exclusively distributed inside the websterite veins[5]; this host mineral is thus a secondary phase of magmatic origin that grew with the entrapment of MI, and not a pre-existing (*i.e.* of residual origin) grain that has re-crystallised in the presence of a pervasively percolating melt. This is an important petrological difference to note in comparison with the inclusions reported by Ionov *et al.*[3].

The vein MI dominantly display the same rounded shapes as those reported by Ionov *et al.*[3], but their host spinel grains are essentially of homogeneous composition at the grain scale (see Fig. 6 in Bénard *et al.*[5]), in line with their secondary, magmatic origin. The systematic distribution of the vein MI in the spinel is as follows: largely crystallised, two-pyroxene MI in the core and clinopyroxene- and/or amphibole-bearing and 'naturally homogeneous' (*i.e.* free of daughter silicate minerals) inclusions from the cores to the rims of spinel (see Figs 5 & 6 in Bénard *et al.*[5]).

Based on the major element compositions of the vein MI and their daughter orthopyroxene and clinopyroxene, it has been shown that these are in equilibrium with calculated Fe–Mg exchange constants (K_D) of 0.28±0.01 for many orthopyroxene-melt pairs and 0.252±0.004 (all 1σ) for clinopyroxene-melt pairs[5]; these values are within the experimental equilibrium range reported in the literature[9]. Given the evidence for equilibrium, pressure estimates of 1.0±0.1 GPa and 0.9±0.1 GPa (all 1σ) were calculated using orthopyroxene-melt and clinopyroxene-melt thermobarometers[9], respectively. These pressure estimates directly support that vein MI were generated by the percolation of a websterite-forming melt in the shallow, sub-arc mantle lithosphere[5] (Fig. 1b).

After heating experiments were conducted by Bénard *et al.*[5] to homogenise all vein MI (*i.e.* dissolve the possibly occurring daughter pyroxenes), it was further established by these authors that the melt compositional fields, both in major and trace elements, formed by these MI could not be related to the pervasive infiltration of the host andesite magma (see Figs 10 & 12 in Bénard *et al.*[5] and Fig. 4a & Supplementary Fig. 1).

All XANES data on vein MI reported in this study for the determination of their S valence state have been acquired on spinel grains that have not been experimentally treated (*i.e.* unheated). Instead, XANES data have been acquired on the 'naturally homogeneous' vein MI from samples Av24 and Av25 (Supplementary Table 1).

In this study, we also report additional spinel-hosted MI in a mantle-derived harzburgite xenolith from the West Bismarck Arc, which petrological and geochemical features are similar to LT inclusions and MP from Kamchatka. The new West Bismarck MI are rounded and disseminated in the spinel harzburgite sample 67-02D(7) originally described by Bénard *et al.*[2,4] and do not show any relationship with a melt channel source (*e.g.* a vein) at the sample scale. Their texture and distribution in harzburgite suggest a formation during the re-crystallisation of

pre-existing (*i.e.* of residual origin) spinel grains in the presence of a pervasively percolating melt, which is very similar to the case of LT inclusions[3] (Fig. 3b).

It has been shown that the petrographic features West Bismarck MI cannot be related to the late-stage percolation of the carrier magmas of the xenoliths at sub-surface conditions, which produces sieve textures[6] (see Figs A1 & A2 in Bénard *et al.*[4]). As for Kamchatka inclusions, the major element compositional range of West Bismarck MI is very different to that of the picrite magmas carrying the xenoliths[2] (Supplementary Fig. 1).

All Raman spectrometry and EPMA data reported in this study for the determination of S and Fe valence states, respectively in West Bismarck MI and their host spinel, have been acquired on spinel grains from sample 67-02D(7) that have not been experimentally treated (*i.e.* unheated). Only West Bismarck MI found 'naturally homogeneous', *i.e.* free of daughter silicate minerals, were investigated here. Bénard *et al.*[2,4] have reported the major element compositions of olivine, orthopyroxene and spinel from sample 67-02D(7), for which equilibrium fO_2 is calculated here (Fig. 1a & Supplementary Table 10). These compositions are given in Supplementary Table 6 (spinel) and Supplementary Table 9 (olivine and orthopyroxene).

Inferences on the nature of the parental melts of all the inclusions have been drawn in earlier studies. From their nature and distribution in spinel, and the presence of similar inclusions in the coexisting olivine and orthopyroxene from the host harzburgite (Fig. 2a), the formation of the spinel-hosted LT inclusions and MP from Kamchatka was interpreted as the result of a multi-stage pervasive percolation process of 'exotic' melts (*i.e.* trapped on their way to the surface and not formed *in situ*) in the sub-arc mantle lithosphere[3]. Ionov *et al.*[3] have originally suggested that LT inclusions formed during the reaction of migrating low-temperature (900–1000°C, based on an experimental treatment for the homogenisation of amphibole-bearing inclusions[3]) andesite-dacite melts (Supplementary Fig. 1) with the surrounding sub-arc mantle lithosphere, which is

indicated for instance by the heterogeneous texture and modified chemistry (generally (Cr, Fe^{3+}, Fe^{2+})-rich with irregular 'halos' and rims) of their host mantle spinel (see Fig. 3 in Ionov *et al.*[3] and Fig. 3a & Supplementary Fig. 7). This indeed suggests the infiltration of an 'exotic' melt in a primary mantle spinel, which also implies that this liquid was potentially out of equilibrium with the residual minerals from the shallow sub-arc mantle lithosphere before being trapped (Supplementary Fig. 1).

After the entrapment of the parental melts of LT inclusions, MP formed in the spinel harzburgites[3]. The MP were likely formed by the percolation of magnesian andesite melts shortly before the capture of Avacha xenoliths in their carrier magmas[3] (Supplementary Fig. 1). Alternatively, MP may have formed by decompression melting at grain boundaries during the ascent of the xenoliths, but this hypothesis cannot be either reconciled with the absence of textural evidence for decompression-driven melting at silicate grain boundaries in sample Av33, nor with the very high volatile contents in the MP glass, indicating mantle conditions[10,11] (see below).

West Bismarck MI display textural features and a major element compositional range close to those of Kamchatka LT inclusions and MP (Fig. 3b & Supplementary Fig. 1); they likely originate from the entrapment of similar melts during a pervasive percolation process in the sub-arc mantle lithosphere as well.

The geochemical features of the vein MI (based on an experimental treatment for the homogenisation of pyroxene-bearing inclusions) indicate that their original parental melts are transitional between SiO_2-rich picrite and high-Ca boninite[5]. These melts formed by high degrees of melting (≥25%) of depleted lherzolite sources fluxed by slab agents at ≤1.5 GPa[5].

The lithophile trace element patterns of glasses in experimentally treated LT inclusions and MP from Kamchatka indicate that their parental melts originally formed by partial melting of, or re-equilibration with, depleted mantle with the ingress of slab agents[3] (Fig. 4a & Supplementary Table 13). The mantle source protoliths (*i.e.* prior to fluxing by slab agents in the

mantle wedge, Fig. 1b) of the inclusion parental melts must be more depleted than DMM, as inferred from the lower abundances of moderately incompatible elements (middle and heavy rare-earth elements and Y) in the inclusion glasses than in MORB[12] (Fig. 4a & Supplementary Table 13). The involvement of slab agents in the formation of the parental melts of Kamchatka LT inclusions and MP is evidenced by elevated abundances of the large-ion lithophile and light rare-earth elements with particularly high U/Th, Pb/Ce and Sr/Nd in the inclusion glasses (Fig. 4a & Supplementary Table 13). Similar features (~15% melt extraction from DMM recorded in their mantle source protoliths with elevated highly incompatible lithophile trace elements) were also deduced for the parental melts of the vein MI (Fig. 4a & Supplementary Table 13).

The glasses in LT inclusions are extremely rich in volatiles (up to ~12 wt% H_2O), which likely originate from slab agents[3,10,11]. The MP glasses have generally high total volatile contents as well[10,11] (up to ~3 wt% H_2O). The vein MI can also contain high H_2O contents up to ~10 wt% (ref. 5). The very high CO_2 concentrations (up to ~1 wt%) sometimes found in the glasses of Kamchatka LT inclusions, MP and vein MI correspond to calculated saturation pressures close to or above 1 GPa[5,10,11], which further indicate that all the inclusions formed in the sub-arc mantle lithosphere. The 'bulk' inclusions (*i.e.* potentially containing immiscible sulfide or anhydrite) where S valence state was characterised in this study contain on average 3400±1100, 250±130 and 1700±600 ppm S (all 1σ), respectively for LT inclusions, vein MI and West Bismarck MI (EPMA data, Supplementary Tables 1 & 3). The 'pure' glasses (*i.e.* free of immiscible sulfide or anhydrite) in LT inclusions contain up to 2000–3000 ppm S (SEM data, Supplementary Table 2), while the sulfide- and anhydrite-free glasses in MP contain 1300±400 ppm S (EPMA data, Supplementary Table 1).

From the XANES data reported in this study, detailed inferences can be made on the elemental distribution and variations in the valence state of sulfur in sub-arc mantle inclusions.

Within LT inclusions and vein MI, S is heterogeneously distributed (Fig. 2b, d & Supplementary Fig. 5a, c). In order to assign XANES features to S valence states of these melt inclusions one has to bear in mind that in magmas, sulfur can be present as S^{2-} and S^{6+} either complexed mainly with Fe, Ni and Cu to form sulfide in the case of S^{2-}, or with O^{2-} to form sulfate in the case of S^{6+} (refs 13,14). Upon sulfide saturation, magmas crystallise either (Fe, Ni, Cu)-S compounds such as mono-sulfide solid solution, pyrrhotite, pentlandite or (chalco-)pyrite containing S^{2-}, or anhydrite ($CaSO_4$) containing S^{6+}. Each S valence state together with the S-coordination produces a distinct feature in the XANES spectrum, which allows discrimination between S^{6+} and S^{2-} dissolved in a glass (*i.e.* a quenched melt) from that incorporated in crystalline sulfide and anhydrite (see ref. 13 for a review).

All XANES spectra in Fig. 2e–g & Supplementary Fig. 5b, d show a peak at 2482.8 eV, which is related to S^{6+} as SO_4^{2-} dissolved in glass. The glass in LT inclusions does not show evidence for S^{2-} and solely contain S^{6+}. Many spectra from S-rich domains in LT inclusions have an additional small peak at ~2486 eV and a broad hump in the energy range of 2490–2510 eV (Fig. 2b, e & Supplementary Fig. 5a, b). These features are characteristic for crystalline anhydrite, the presence of which is further confirmed by Raman spectrometry (Figs 2h, i & 3c), EPMA and SEM imaging and EDS spectra (Supplementary Figs 2–4).

In addition to S^{6+} as SO_4^{2-} dissolved in glass, the vein MI contain S^{2-} evidenced by a peak at 2472.5 eV (Fig. 2g & Supplementary Fig. 5d). This is in the energy range where crystalline sulfide such as pyrrhotite and pyrite have their most prominent XANES signals[13,14]. The peak at 2472.5 eV also varies in intensity and is most prominent in XANES spectra from areas with high S concentrations ($0.24 \leq S^{6+}/\sum S \leq 0.44$), while it only appears as a hump in the background of the other XANES spectra ($0.62 \leq S^{6+}/\sum S \leq 0.88$, Fig. 2d, g & Supplementary Fig. 5c, d). Thus, S^{6+} from the glass and S^{2-} from the crystalline sulfide contributed to the XANES spectra from vein MI with $0.24 \leq S^{6+}/\sum S \leq 0.44$. For those with higher $0.62 \leq S^{6+}/\sum S \leq 0.88$, which were acquired on glass with

more homogeneous S composition, they likely reflect the presence of both dissolved S^{2-} and S^{6+} in the glass as for MP (Fig. 2c–g & Supplementary Fig. 5c, d).

A high photon flux during XANES measurements may cause beam damage affecting S valence states in glasses[13–15]. Indeed, S^{4+} is visible in some spectra with a peak at 2478 eV (Fig. 2e–g & Supplementary Fig. 5b, d). As the beam damage only affects glasses and has not been observed in solid S-bearing compounds[13–15], it is confirmed that XANES spectra from areas with low S concentrations for all inclusions predominantly represent glass analyses (Fig. 2b–g & Supplementary Fig. 5). Beam damage can also affect the calculated $S^{6+}/\sum S$ as S^{4+} is produced by photo-reduction of S^{6+} in glasses[13–15]. Therefore, the calculated $S^{6+}/\sum S$ are minima for each glass measurement. The effect of beam damage on the calculated $S^{6+}/\sum S$ is estimated to be in the range of 0.05 to 0.1.

From the EPMA data reported in this study, more inferences can be made on the entrapment processes of LT inclusions and West Bismarck MI, as well as redox coupling during melt-rock interactions in the sub-arc mantle lithosphere. Partially reacted spinel grains show that the composition of this mineral is modified (*e.g*. relatively enriched in Cr, Fe^{3+} and Fe^{2+}, see the core to rim profile in Supplementary Table 5) along areas selectively intruded by melts (inclusion-filled 'trails' or rims, see Fig. 2 in Ionov *et al.*[3] and Supplementary Fig. 7). These textural and chemical features record the ingress of a dissolution and re-precipitation process of spinel during melt-rock interactions in the sub-arc mantle lithosphere, which can eventually affect spinel grains entirely (Figs 2 & 3 in Ionov *et al.*[3] and Figs 2a & 3a, b).

In addition, there is commonly an increase in the $Fe^{3+}/\sum Fe$ of spinel closely surrounding LT inclusions and West Bismarck MI ('halos', Fig. 3d, Supplementary Tables 5 & 6). A similar observation is made in spinel halos surrounding MP using the profile data reported by Ionov *et al.*[3] (their Fig. 3) and uncorrected spinel stoichiometric calculations (Supplementary Fig. 8 &

Supplementary Table 8). However, we note that the halos around LT inclusions and West Bismarck MI tend to be very irregular (Fig. 3d). This suggests that these variations are unlikely formed by simple post-entrapment crystallization of spinel from the melt on the walls of the inclusions during re-precipitation (possibly accompanied by re-equilibration through solid-state diffusion); these processes are instead characterised by symmetrical composition profiles, for instance around phenocryst-hosted inclusions in magmas[16]. Furthermore, if the re-precipitated spinel is generally enriched in both Fe^{3+} and Fe^{2+}, the halos tend to be depleted in Fe^{2+} (Supplementary Tables 5 & 6). In an Cr# *vs*. Mg# compositional space (with Cr# stands for Cr/(Cr+Al)), the effects of dissolution and re-precipitation reactions on spinel compositions are thus characterised by an increase in Cr# and decrease in Mg#, whereas an opposite trend is observed for halo formation (see Fig. 3 in Ionov *et al.*[3]). This records two distinct processes, with the second event (*i.e.* halo formation) occurring at a post-entrapment stage.

From the typical variations in spinel composition in halos around LT inclusions and West Bismarck MI (Supplementary Tables 5 & 6), the exchange reactions between a (S^{6+}, H_2O)-bearing melt and spinel can be summarised as follows:

$$[MgO]_{melt}+[FeO]_{spinel}=>[MgO]_{spinel}+[FeO]_{melt} \quad (1)$$

$$9[FeO]_{melt}+[H_2SO_4]_{melt}=>FeS+[H_2O]_{melt}+4[Fe_2O_3]_{melt} \quad (2)$$

$$[Fe_2O_3]_{melt}+[Cr_2O_3]_{spinel}=>[Fe_2O_3]_{spinel}+[Cr_2O_3]_{melt} \quad (3)$$

$$[Al_2O_3]_{melt}+[Cr_2O_3]_{spinel}=>[Al_2O_3]_{spinel}+[Cr_2O_3]_{melt} \quad (4)$$

Reaction (2) must be the first to operate during or after melt entrapment because Fe^{2+}–Mg equilibration is very fast in many mineral-melt systems, including those with spinel[17]. Given that the equilibrium constant for reaction (2) is strongly temperature-dependant[17,18], magmatic spinel-hosted inclusions sometimes show a trend of decreasing Mg# in the inclusion glass at constant Mg# in host spinel[19]. However, the situation for inclusions formed in the sub-arc mantle shortly before eruption[20] may be very distinct from those residing in magma chambers for longer periods

of time. In particular, the temperature effect on re-equilibration during cooling is likely more limited; it could even be insignificant in some cases, such as for LT inclusions formed at 900–1000°C (as inferred from homogenisation experiments[3]), which is very close to the equilibration temperatures of their host harzburgites in the Kamchatka sub-arc mantle lithosphere[1,2]. Therefore, the more limited variations in Mg# in spinel than in the coexisting inclusion glasses (*e.g.* 0.63–0.69 in MP for 0.54–0.57 in spinel, Supplementary Tables 1 & 5) suggests that the transfer of small amounts of Fe^{2+} from the host spinel into the included melts most likely occurred because of post-entrapment re-equilibration of spinel-melt pairs to slightly lower temperature conditions. This process ultimately conducted to the formation of irregular halos.

In a scenario where LT inclusions and West Bismarck MI result of a dissolution and re-precipitation process of mantle spinel by SiO_2-rich parental melts, both phases should tend to reach redox equilibrium when the inclusions form. It appears to be the case for most of the inclusions in this study, if one estimates their fO_2 using the $S^{6+}/\sum S$ equilibrium curve for basalt-andesite silicate melts for low-pressure (≤0.2 GPa) conditions[21–23] (Fig. 1a). This is confirmed by the calculated $Fe^{3+}/\sum Fe$ in spinel, based on the $Fe^{3+}/\sum Fe$ equilibrium in silicate melts[24,25] and partitioning coefficients for Fe^{3+} and Fe^{2+} between Cr-bearing spinel and melt[26] (Fig. 1a & Supplementary Table 12). However, it is important to note that saturation in anhydrite in West Bismarck MI (Figs 2i, 3c & Supplementary Table 3) may correspond to higher fO_2 conditions (by ≥0.5 log unit) than those calculated using oxybarometry[27,28] and the stable composition of spinel in the percolated harzburgite samples (Fig. 1a). This suggests that a certain extent of redox disequilibrium may have existed between the percolating inclusion parental melts and the harzburgites before interactions through dissolution and re-precipitation took place, but also that this disequilibrium was partly preserved after entrapment for some inclusions. The preservation of a certain extent of disequilibrium is likely related to the specific mechanisms of pervasive melt

percolation in mantle peridotites, which commonly involve kinetic fractionation with only partial re-equilibration of melts and rocks[29].

High fO_2 conditions (ranging 1–1.5 log units above FMQ) recorded by the percolated harzburgites in this study likely result of a redox process by the S^{6+}-bearing parental melts of LT inclusions and West Bismarck MI following reaction (2), or a similar one (Fig. 1a). Such process is consistent with the unusually high oxidation states of the percolated harzburgite samples in this study[2,4]. The difference in atomic composition between the irregular halos and the flat portions of the EPMA profiles show that Fe^{2+}–Mg^{2+} and (Fe^{3+}, Al^{3+})–Cr^{3+} cation substitutions in reactions (1), (3) and (4) are balanced around LT inclusions and West Bismarck MI, within the error of the Wood & Virgo[30] correction method (*e.g.* ±0.025 on the corrected $Fe^{3+}/\sum Fe$ in spinel, Supplementary Tables 5 & 6). Similarly, very similar amounts of Fe^{3+} and Fe^{2+} are calculated to respectively depart and enter the inclusions (0.01–0.02 At%, Supplementary Tables 5 & 6), which indicates a nearly equimolar Fe^{3+}–Fe^{2+} exchange between the host spinel and the inclusions at a post-entrapment stage. This suggests that it is the progress of reaction (2), or a similar one, which has resulted in the increase of $Fe^{3+}/\sum Fe$ in the host spinel halos because of the availability of $[FeO]_{melt}$ from reaction (1) and the presence of $[H_2SO_4]_{melt}$. An alternative process, for instance involving significant Fe oxidation in the MI due to a prolonged post-entrapment exchange with the host spinel at constant fO_2 and decreasing temperature (as previously reported on phenocryst-hosted inclusions in magmas[19]), cannot be reconciled with the stoichiometry of the cation exchanges derived from the profiles (*e.g.* Fe^{3+} in the included melts does not significantly exchange with Al and Cr in sub-arc mantle spinel[19], Supplementary Tables 5 & 6). Instead, the observed increase in $Fe^{3+}/\sum Fe$ in the host spinel halos (Fig. 3d, Supplementary Fig. 7, Supplementary Tables 5 & 6) can only be explained by the presence of an oxidising component in the percolating parental melts of the inclusions. The progress of reaction (2) is further consistent with the possible, but importantly, not systematic occurrence of immiscible sulfides in LT

inclusions (Supplementary Figs 2–4, 6 & 7) and the only slight decrease of $S^{6+}/\sum S$ from unity in low-S areas of LT inclusions (Fig. 2b, e & Supplementary Fig. 5).

To sum up, both the oxidised nature of sub-arc mantle harzburgite samples modified by dissolution and re-precipitation processes, and the Fe^{3+}-rich nature of the post-entrapment halos around inclusions, record the strong oxidising power of SiO_2- and S^{6+}-rich silicate melts commonly percolating the sub-arc mantle.

Supplementary References

1. Ionov, D. A. Petrology of mantle wedge lithosphere: New data on supra-subduction zone peridotite xenoliths from the andesitic Avacha volcano, Kamchatka. *J. Petrol.* **51**, 327–361 (2010).
2. Bénard, A., Arculus, R. J., Nebel, O., Ionov D. A. & McAlpine S. R. B. Silica-enriched mantle sources of subalkaline picrite-boninite-andesite island arc magmas. *Geochim. Cosmochim. Acta* **199**, 287–303 (2017).
3. Ionov, D. A., Bénard, A. & Plechov, P. Y. Melt evolution in subarc mantle: evidence from heating experiments on spinel-hosted melt inclusions in peridotite xenoliths from the andesitic Avacha volcano (Kamchatka, Russia). *Contrib. Mineral. Petrol.* **162**, 1159–1174 (2011).
4. Bénard, A., Woodland, A. B., Arculus, R. J., Nebel, O. & McAlpine, S. R. B. Variation in sub-arc mantle oxygen fugacity during partial melting recorded in refractory peridotite xenoliths from the West Bismarck Arc. *Chem. Geol.* **160**, 16–30 (2018).
5. Bénard, A. *et al.* Primary silica-rich picrite and high-Ca boninite melt inclusions in pyroxenite veins from the Kamchatka sub-arc mantle. *J. Petrol.* **57**, 1955–1982 (2016).
6. Shaw, C. S. J. & Dingwell, D. B. Experimental peridotite-melt reaction at one atmosphere: a textural and chemical study. *Contrib. Mineral. Petrol.* **155**, 199–214 (2008).

7. Bénard, A. & Ionov, D. A. Melt- and fluid-rock interaction in supra-subduction lithospheric mantle: Evidence from andesite-hosted veined peridotite xenoliths. *J. Petrol.* **54**, 2339–2378 (2013).

8. Halama, R., Savov, I. P., Rudnick, R. L. & McDonough, W. F. Insights into Li and Li isotope cycling and sub-arc metasomatism from veined mantle xenoliths, Kamchatka. *Contrib. Mineral. Petrol.* **158**, 197–222 (2009).

9. Putirka, K. D. Thermometers and barometers for volcanic systems. *Rev. Mineral. Geochem.*, **69**, 61–120 (2008).

10. Bénard, A., Ionov, D. A., Shimizu, N. & Plechov, P. Y. The volatile content of subduction zone melts and fluids. *Min. Mag.* **75**, 513 (2011).

11. Bénard, A., Shimizu, N., Klimm, K., Ionov, D. A. & Plechov, P. Y. Volatile content, S partitioning, speciation and isotopes in the mantle wedge. *European Mineralogical Conference* **1**, EMC2012-582-3 (2012).

12. Salters, V. J. M. & Stracke, A. Composition of the depleted mantle. *Geochem. Geophys. Geosyst.* **5**, doi: 10.1029/2003GC000597 (2004).

13. Wilke, M., Klimm, K. & Kohn, S. C. Spectroscopic studies on sulfur speciation in synthetic and natural glasses. *Rev. Mineral. Geochem.* **73**, 41–78 (2011).

14. Klimm, K., Kohn, S. C., O'Dell, L. A., Botcharnikov, R. E. & Smith, M. E. The dissolution mechanism of sulphur in hydrous silicate melts. I: Assessment of analytical techniques in determining the sulphur speciation in iron-free to iron-poor glasses. *Chem. Geol.* **322-323**, 237–249 (2012).

15. Wilke, M. *et al.* The origin of S^{4+} detected in silicate glasses by XANES. *Am. Min.* **93**, 235–240 (2008).

16. Danyushevsky, L. D., Della-Pasqua, F. N. & Sokolov, S. Re-equilibration of melt inclusions trapped by magnesian olivine phenocrysts from subduction-related magmas: petrological implications. *Contrib. Mineral. Petrol.* **138**, 68–83 (2000).

17. Kamenetsky, V. S., Crawford, A. J. & Meffre, S. Factors controlling chemistry of magmatic spinel: an empirical study of associated olivine, Cr-spinel and melt inclusions from primitive rocks. *J. Petrol.* **42**, 655–671 (2001).

18. Nikolaev, G. S., Ariskin, A. A. & Barmina G. S. SPINMELT-2.0: Simulation of spinel-melt equilibrium in basaltic systems under pressures up to 15 kbar: I. Model formulation, calibration, and tests. *Geochem. Int.* **56**, 24–45 (2018).

19. Portnyagin, M., Hoernle, K. & Savelyev, D. Ultra-depleted melts from Kamchatkan ophiolites: Evidence for the interaction of the Hawaiian plume with an oceanic spreading center in the Cretaceous? *Earth Planet. Sci. Lett.* **287**, 194–204 (2009).

20. Tollan, P. M. E., O'Neill, H. St. C., Hermann, J., Benedictus, A. & Arculus, R. J. Frozen melt-rock reaction in a peridotite xenolith from sub-arc mantle recorded by diffusion of trace elements and water olivine. *Earth Planet. Sci. Lett.* **422**, 169–181 (2015).

21. Jugo, P. J., Wilke, M. & Botcharnikov, R. E. Sulfur K-edge XANES analysis of natural and synthetic basaltic glasses: Implications for S speciation and S content as function of oxygen fugacity. *Geochim. Cosmochim. Acta* **74**, 5926–5938 (2010).

22. Klimm, K., Kohn, S. C. & Botcharnikov, R. E. The dissolution mechanism of sulphur in hydrous silicate melts. II: Solubility and speciation of sulphur in hydrous silicate melts as a function of fO_2. *Chem. Geol.* **322-323**, 250–267 (2012).

23. Botcharnikov, R. E. *et al.* High gold concentrations in sulphide-bearing magma under oxidizing conditions. *Nat. Geosci.* **4**, 112–115 (2011).

24. Kress, V. C. & Carmichael, I. S. E. The compressibility of silicate liquids containing Fe_2O_3 and the effect of composition, temperature, oxygen fugacity and pressure on their redox states. *Contrib. Mineral. Petrol.* **108**, 82–92 (1991).

25. Ghiorso, M. S., Hirschmann M. M., Reiners, P. W. & Kress, V. C. The pMELTS: A revision of MELTS for improved calculations of phase relations and major element partitioning related to partial melting of the mantle to 3 GPa. *Geochem. Geophys. Geosyst.* **3**, doi: 10.1029/2001GC000217 (2002).

26. Mallmann, G. & O'Neill, H. St. C. The crystal/melt partitioning of V during mantle melting as a function of oxygen fugacity compared with some other elements (Al, P, Ca, Sc, Ti, Cr, Fe, Ga, Y, Zr and Nb). *J. Petrol.* **50**, 1765–1794 (2009).

27. Wood, B. J., Bryndzia, L. T. & Johnson, K. E. Mantle oxidation state and its relationship to tectonic environment and fluid speciation. *Science* **248**, 337–345 (1990).

28. Ballhaus, C., Berry, R. F. & Green, D. H. High-pressure calibration of the olivine-orthopyroxene-spinel oxygen geobarometer: implications for the oxidation state of the upper mantle. *Contrib. Mineral. Petrol.* **107**, 27–40 (1991).

29. Ionov, D. A., Bodinier, J.-L., Mukasa, S. B. & Zanetti, A. Mechanisms and sources of mantle metasomatism: major and trace element compositions of peridotite xenoliths from Spitsbergen in the context of numerical modelling. *J. Petrol.* **43**, 2219–2259 (2002).

30. Wood, B. J. & Virgo, D. Upper mantle oxidation state: Ferric iron contents of lherzolite spinels by ^{57}Fe Mössbauer spectroscopy and resultant oxygen fugacities. *Geochim. Cosmochim. Acta* **53**, 1277–1291 (1989).

31. Fiege, A. *et al.*, Sulfur isotope fractionation between fluid and andesitic melt: An experimental study. *Geochim. Cosmochim. Acta* **142**, 501–521 (2014).
